# The evolution of non-small cell lung cancer metastases in TRACERx

**DOI:** 10.1038/s41586-023-05729-x

**Published:** 2023-04-12

**Authors:** Maise Al Bakir, Ariana Huebner, Carlos Martínez-Ruiz, Kristiana Grigoriadis, Thomas B. K. Watkins, Oriol Pich, David A. Moore, Selvaraju Veeriah, Sophia Ward, Joanne Laycock, Diana Johnson, Andrew Rowan, Maryam Razaq, Mita Akther, Cristina Naceur-Lombardelli, Paulina Prymas, Antonia Toncheva, Sonya Hessey, Michelle Dietzen, Emma Colliver, Alexander M. Frankell, Abigail Bunkum, Emilia L. Lim, Takahiro Karasaki, Christopher Abbosh, Crispin T. Hiley, Mark S. Hill, Daniel E. Cook, Gareth A. Wilson, Roberto Salgado, Emma Nye, Richard Kevin Stone, Dean A. Fennell, Gillian Price, Keith M. Kerr, Babu Naidu, Gary Middleton, Yvonne Summers, Colin R. Lindsay, Fiona H. Blackhall, Judith Cave, Kevin G. Blyth, Arjun Nair, Asia Ahmed, Magali N. Taylor, Alexander James Procter, Mary Falzon, David Lawrence, Neal Navani, Ricky M. Thakrar, Sam M. Janes, Dionysis Papadatos-Pastos, Martin D. Forster, Siow Ming Lee, Tanya Ahmad, Sergio A. Quezada, Karl S. Peggs, Peter Van Loo, Caroline Dive, Allan Hackshaw, Nicolai J. Birkbak, Simone Zaccaria, TRACERx Consortium, Maise Al Bakir, Ariana Huebner, Carlos Martínez-Ruiz, Kristiana Grigoriadis, Thomas B. K. Watkins, Oriol Pich, David A. Moore, Selvaraju Veeriah, Sophia Ward, Andrew Rowan, Cristina Naceur-Lombardelli, Paulina Prymas, Antonia Toncheva, Sonya Hessey, Michelle Dietzen, Emma Colliver, Alexander M. Frankell, Abigail Bunkum, Emilia L. Lim, Takahiro Karasaki, Christopher Abbosh, Crispin T. Hiley, Mark S. Hill, Gareth A. Wilson, Roberto Salgado, Emma Nye, Richard Kevin Stone, Dean A. Fennell, Gillian Price, Keith M. Kerr, Babu Naidu, Gary Middleton, Yvonne Summers, Colin R. Lindsay, Fiona H. Blackhall, Judith Cave, Kevin G. Blyth, Arjun Nair, Asia Ahmed, Magali N. Taylor, Alexander James Procter, Mary Falzon, David Lawrence, Neal Navani, Ricky M. Thakrar, Sam M. Janes, Dionysis Papadatos-Pastos, Martin D. Forster, Siow Ming Lee, Tanya Ahmad, Sergio A. Quezada, Karl S. Peggs, Peter Van Loo, Caroline Dive, Allan Hackshaw, Nicolai J. Birkbak, Simone Zaccaria, Jason F. Lester, Amrita Bajaj, Apostolos Nakas, Azmina Sodha-Ramdeen, Keng Ang, Mohamad Tufail, Mohammed Fiyaz Chowdhry, Molly Scotland, Rebecca Boyles, Sridhar Rathinam, Claire Wilson, Domenic Marrone, Sean Dulloo, Gurdeep Matharu, Jacqui A. Shaw, Joan Riley, Lindsay Primrose, Ekaterini Boleti, Heather Cheyne, Mohammed Khalil, Shirley Richardson, Tracey Cruickshank, Sarah Benafif, Kayleigh Gilbert, Akshay J. Patel, Aya Osman, Christer Lacson, Gerald Langman, Helen Shackleford, Madava Djearaman, Salma Kadiri, Angela Leek, Jack Davies Hodgkinson, Nicola Totten, Angeles Montero, Elaine Smith, Eustace Fontaine, Felice Granato, Helen Doran, Juliette Novasio, Kendadai Rammohan, Leena Joseph, Paul Bishop, Rajesh Shah, Stuart Moss, Vijay Joshi, Philip Crosbie, Fabio Gomes, Kate Brown, Mathew Carter, Anshuman Chaturvedi, Lynsey Priest, Pedro Oliveira, Matthew G. Krebs, Alexandra Clipson, Jonathan Tugwood, Alastair Kerr, Dominic G. Rothwell, Elaine Kilgour, Hugo J. W. L. Aerts, Roland F. Schwarz, Tom L. Kaufmann, Rachel Rosenthal, Zoltan Szallasi, Judit Kisistok, Mateo Sokac, Miklos Diossy, Jonas Demeulemeester, Aengus Stewart, Alastair Magness, Angeliki Karamani, Benny Chain, Brittany B. Campbell, Carla Castignani, Chris Bailey, Clare Puttick, Clare E. Weeden, Claudia Lee, Corentin Richard, David R. Pearce, Despoina Karagianni, Dhruva Biswas, Dina Levi, Elena Hoxha, Elizabeth Larose Cadieux, Eva Grönroos, Felip Gálvez-Cancino, Foteini Athanasopoulou, Francisco Gimeno-Valiente, George Kassiotis, Georgia Stavrou, Gerasimos Mastrokalos, Haoran Zhai, Helen L. Lowe, Ignacio Matos, Jacki Goldman, James L. Reading, James R. M. Black, Javier Herrero, Jayant K. Rane, Jerome Nicod, Jie Min Lam, John A. Hartley, Katey S. S. Enfield, Kayalvizhi Selvaraju, Kerstin Thol, Kevin Litchfield, Kevin W. Ng, Kezhong Chen, Krijn Dijkstra, Krupa Thakkar, Leah Ensell, Mansi Shah, Marcos Vasquez, Maria Litovchenko, Mariana Werner Sunderland, Michelle Leung, Mickael Escudero, Mihaela Angelova, Miljana Tanić, Monica Sivakumar, Nnennaya Kanu, Olga Chervova, Olivia Lucas, Othman Al-Sawaf, Philip Hobson, Piotr Pawlik, Robert Bentham, Robert E. Hynds, Roberto Vendramin, Sadegh Saghafinia, Saioa López, Samuel Gamble, Seng Kuong Anakin Ung, Sharon Vanloo, Stefan Boeing, Stephan Beck, Supreet Kaur Bola, Tamara Denner, Teresa Marafioti, Thanos P. Mourikis, Victoria Spanswick, Vittorio Barbè, Wei-Ting Lu, William Hill, Wing Kin Liu, Yin Wu, Yutaka Naito, Zoe Ramsden, Catarina Veiga, Gary Royle, Charles-Antoine Collins-Fekete, Francesco Fraioli, Paul Ashford, Tristan Clark, Elaine Borg, James Wilson, Davide Patrini, Emilie Martinoni Hoogenboom, Fleur Monk, James W. Holding, Junaid Choudhary, Kunal Bhakhri, Marco Scarci, Martin Hayward, Nikolaos Panagiotopoulos, Pat Gorman, Reena Khiroya, Robert C. M. Stephens, Yien Ning Sophia Wong, Steve Bandula, Abigail Sharp, Sean Smith, Nicole Gower, Harjot Kaur Dhanda, Kitty Chan, Camilla Pilotti, Rachel Leslie, Anca Grapa, Hanyun Zhang, Khalid AbdulJabbar, Xiaoxi Pan, Yinyin Yuan, David Chuter, Mairead MacKenzie, Serena Chee, Aiman Alzetani, Lydia Scarlett, Jennifer Richards, Papawadee Ingram, Silvia Austin, Eric Lim, Paulo De Sousa, Simon Jordan, Alexandra Rice, Hilgardt Raubenheimer, Harshil Bhayani, Lyn Ambrose, Anand Devaraj, Hema Chavan, Sofina Begum, Silviu I. Buderi, Daniel Kaniu, Mpho Malima, Sarah Booth, Andrew G. Nicholson, Nadia Fernandes, Pratibha Shah, Chiara Proli, Madeleine Hewish, Sarah Danson, Michael J. Shackcloth, Lily Robinson, Peter Russell, Craig Dick, John Le Quesne, Alan Kirk, Mo Asif, Rocco Bilancia, Nikos Kostoulas, Mathew Thomas, Mariam Jamal-Hanjani, Nicholas McGranahan, Charles Swanton, Mariam Jamal-Hanjani, Nicholas McGranahan, Charles Swanton

**Affiliations:** 1 Cancer Research UK Lung Cancer Centre of Excellence, University College London Cancer Institute, London, UK; 2 Cancer Evolution and Genome Instability Laboratory, The Francis Crick Institute, London, UK; 3 Cancer Genome Evolution Research Group, Cancer Research UK Lung Cancer Centre of Excellence, University College London Cancer Institute, London, UK; 4 Department of Cellular Pathology, University College London Hospitals, London, UK; 5 Advanced Sequencing Facility, The Francis Crick Institute, London, UK; 6 Cancer Metastasis Laboratory, University College London Cancer Institute, London, UK; 7 Computational Cancer Genomics Research Group, University College London Cancer Institute, London, UK; 8 Department of Pathology, ZAS Hospitals, Antwerp, Belgium; 9 Division of Research, Peter MacCallum Cancer Centre, Melbourne, Victoria Australia; 10 Experimental Histopathology, The Francis Crick Institute, London, UK; 11 University of Leicester, Leicester, UK; 12 University Hospitals of Leicester NHS Trust, Leicester, UK; 13 Department of Medical Oncology, Aberdeen Royal Infirmary NHS Grampian, Aberdeen, UK; 14 University of Aberdeen, Aberdeen, UK; 15 Department of Pathology, Aberdeen Royal Infirmary NHS Grampian, Aberdeen, UK; 16 Birmingham Acute Care Research Group, Institute of Inflammation and Ageing, University of Birmingham, Birmingham, UK; 17 University Hospital Birmingham NHS Foundation Trust, Birmingham, UK; 18 Institute of Immunology and Immunotherapy, University of Birmingham, Birmingham, UK; 19 Division of Cancer Sciences, The University of Manchester and The Christie NHS Foundation Trust, Manchester, UK; 20 Department of Oncology, University Hospital Southampton NHS Foundation Trust, Southampton, UK; 21 School of Cancer Sciences, University of Glasgow, Glasgow, UK; 22 Cancer Research UK Beatson Institute, Glasgow, UK; 23 Queen Elizabeth University Hospital, Glasgow, UK; 24 Department of Radiology, University College London Hospitals, London, UK; 25 UCL Respiratory, Department of Medicine, University College London, London, UK; 26 Department of Thoracic Surgery, University College London Hospital NHS Trust, London, UK; 27 Lungs for Living Research Centre, UCL Respiratory, University College London, London, UK; 28 Department of Thoracic Medicine, University College London Hospitals, London, UK; 29 Department of Oncology, University College London Hospitals, London, UK; 30 Immune Regulation and Tumour Immunotherapy Group, Cancer Immunology Unit, Research Department of Haematology, University College London Cancer Institute, London, UK; 31 Department of Haematology, University College London Hospitals, London, UK; 32 Cancer Immunology Unit, Research Department of Haematology, University College London Cancer Institute, London, UK; 33 Cancer Genomics Laboratory, The Francis Crick Institute, London, UK; 34 Department of Genetics, The University of Texas MD Anderson Cancer Center, Houston, TX USA; 35 Department of Genomic Medicine, The University of Texas MD Anderson Cancer Center, Houston, TX USA; 36 Cancer Research UK Manchester Institute Cancer Biomarker Centre, University of Manchester, Manchester, UK; 37 Cancer Research UK Lung Cancer Centre of Excellence, University of Manchester, Manchester, UK; 38 Cancer Research UK & UCL Cancer Trials Centre, London, UK; 39 Department of Molecular Medicine, Aarhus University Hospital, Aarhus, Denmark; 40 Department of Clinical Medicine, Aarhus University, Aarhus, Denmark; 41 Bioinformatics Research Centre, Aarhus University, Aarhus, Denmark; 42 Singleton Hospital, Swansea Bay University Health Board, Swansea, UK; 43 Cancer Research Centre, University of Leicester, Leicester, UK; 44 Royal Free Hospital, Royal Free London NHS Foundation Trust, London, UK; 45 Aberdeen Royal Infirmary NHS Grampian, Aberdeen, UK; 46 The Whittington Hospital NHS Trust, London, UK; 47 Manchester Cancer Research Centre Biobank, Manchester, UK; 48 Wythenshawe Hospital, Manchester University NHS Foundation Trust, Wythenshawe, UK; 49 Division of Infection, Immunity and Respiratory Medicine, University of Manchester, Manchester, UK; 50 The Christie NHS Foundation Trust, Manchester, UK; 51 Artificial Intelligence in Medicine (AIM) Program, Mass General Brigham, Harvard Medical School, Boston, MA USA; 52 Department of Radiation Oncology, Brigham and Women’s Hospital, Dana-Farber Cancer Institute, Harvard Medical School, Boston, MA USA; 53 Radiology and Nuclear Medicine, CARIM & GROW, Maastricht University, Maastricht, The Netherlands; 54 Institute for Computational Cancer Biology, Center for Integrated Oncology (CIO), Cancer Research Center Cologne Essen (CCCE), Faculty of Medicine and University Hospital Cologne, University of Cologne, Cologne, Germany; 55 Berlin Institute for the Foundations of Learning and Data (BIFOLD), Berlin, Germany; 56 Berlin Institute for Medical Systems Biology, Max Delbrück Center for Molecular Medicine in the Helmholtz Association (MDC), Berlin, Germany; 57 Danish Cancer Society Research Center, Copenhagen, Denmark; 58 Computational Health Informatics Program, Boston Children’s Hospital, Boston, MA USA; 59 Department of Bioinformatics, Semmelweis University, Budapest, Hungary; 60 Department of Physics of Complex Systems, ELTE Eötvös Loránd University, Budapest, Hungary; 61 Integrative Cancer Genomics Laboratory, Department of Oncology, KU Leuven, Leuven, Belgium; 62 VIB—KU Leuven Center for Cancer Biology, Leuven, Belgium; 63 The Francis Crick Institute, London, UK; 64 University College London Cancer Institute, London, UK; 65 Medical Genomics, University College London Cancer Institute, London, UK; 66 Bill Lyons Informatics Centre, University College London Cancer Institute, London, UK; 67 Retroviral Immunology Group, The Francis Crick Institute, London, UK; 68 Department of Infectious Disease, Faculty of Medicine, Imperial College London, London, UK; 69 Tumour Immunogenomics and Immunosurveillance Laboratory, University College London Cancer Institute, London, UK; 70 Department of Molecular Oncology and Immunology, The Netherlands Cancer Institute, Amsterdam, The Netherlands; 71 Oncode Institute, Utrecht, The Netherlands; 72 Experimental Oncology, Institute for Oncology and Radiology of Serbia, Belgrade, Serbia; 73 Centre for Medical Image Computing, Department of Medical Physics and Biomedical Engineering, University College London, London, UK; 74 Department of Medical Physics and Bioengineering, University College London Cancer Institute, London, UK; 75 Department of Medical Physics and Biomedical Engineering, University College London, London, UK; 76 Institute of Nuclear Medicine, Division of Medicine, University College London, London, UK; 77 Institute of Structural and Molecular Biology, University College London, London, UK; 78 University College London, London, UK; 79 University College London Hospitals, London, UK; 80 The Institute of Cancer Research, London, UK; 81 The University of Texas MD Anderson Cancer Center, Houston, TX USA; 82 Independent Cancer Patients’ Voice, London, UK; 83 University Hospital Southampton NHS Foundation Trust, Southampton, UK; 84 Academic Division of Thoracic Surgery, Imperial College London, London, UK; 85 Royal Brompton and Harefield Hospitals, Guy’s and St Thomas’ NHS Foundation Trust, London, UK; 86 Department of Histopathology, Royal Brompton and Harefield Hospitals, Guy’s and St Thomas’ NHS Foundation Trust, London, UK; 87 National Heart and Lung Institute, Imperial College London, London, UK; 88 Royal Surrey Hospital, Royal Surrey Hospitals NHS Foundation Trust, Guilford, UK; 89 University of Surrey, Guilford, UK; 90 Sheffield Teaching Hospitals NHS Foundation Trust, Sheffield, UK; 91 Liverpool Heart and Chest Hospital, Liverpool, UK; 92 Princess Alexandra Hospital, The Princess Alexandra Hospital NHS Trust, Harlow, UK; 93 NHS Greater Glasgow and Clyde, Glasgow, UK; 94 Pathology Department, Queen Elizabeth University Hospital, NHS Greater Glasgow and Clyde, Glasgow, UK; 95 Golden Jubilee National Hospital, Clydebank, UK

**Keywords:** Computational biology and bioinformatics, Evolutionary genetics, Cancer genomics, Metastasis, Non-small-cell lung cancer

## Abstract

Metastatic disease is responsible for the majority of cancer-related deaths^1^. We report the longitudinal evolutionary analysis of 126 non-small cell lung cancer (NSCLC) tumours from 421 prospectively recruited patients in TRACERx who developed metastatic disease, compared with a control cohort of 144 non-metastatic tumours. In 25% of cases, metastases diverged early, before the last clonal sweep in the primary tumour, and early divergence was enriched for patients who were smokers at the time of initial diagnosis. Simulations suggested that early metastatic divergence more frequently occurred at smaller tumour diameters (less than 8 mm). Single-region primary tumour sampling resulted in 83% of late divergence cases being misclassified as early, highlighting the importance of extensive primary tumour sampling. Polyclonal dissemination, which was associated with extrathoracic disease recurrence, was found in 32% of cases. Primary lymph node disease contributed to metastatic relapse in less than 20% of cases, representing a hallmark of metastatic potential rather than a route to subsequent recurrences/disease progression. Metastasis-seeding subclones exhibited subclonal expansions within primary tumours, probably reflecting positive selection. Our findings highlight the importance of selection in metastatic clone evolution within untreated primary tumours, the distinction between monoclonal versus polyclonal seeding in dictating site of recurrence, the limitations of current radiological screening approaches for early diverging tumours and the need to develop strategies to target metastasis-seeding subclones before relapse.

## Abstract (web summary)

A longitudinal evolutionary analysis of 126 lung cancer patients with metastatic disease reveals the timing of metastatic divergence, modes of dissemination and the genomic events subject to selection during the metastatic transition.

## Main

Primary lung cancer (80% of which is of the non-small cell lung cancer (NSCLC) histological subtype^2^) is the leading cause of cancer-related mortality worldwide. The majority of deaths occur in patients with metastatic disease^1^. A better understanding of the metastatic process is needed to guide therapeutic strategies and improve patient outcomes.

Our ability to explore the process of metastasis may be limited by patient recruitment bias, small patient sample sizes, heterogeneous treatment histories, limited follow-up and inadequate tumour sampling. The TRACERx study^3^ (TRAcking non-small cell lung Cancer Evolution through therapy (Rx); ClinicalTrials.gov: NCT01888601) aimed to address these limitations through prospective enrolment of patients with early-stage (I–III) untreated NSCLC. Multiple regions from primary and metastatic NSCLCs are sampled and patients are followed-up over 5 years through the adjuvant setting to cure or recurrence. TRACERx reflects real-world clinical presentations across the UK treated in a universal healthcare system across 19 hospital sites between 2014 and 2021.

Using whole-exome sequencing (WES), we investigated the timing and pattern of metastatic dissemination, and whether platinum chemotherapy affects tumour evolution. We explored selection in metastasizing and non-metastasizing subclones and examined the impact of tissue sampling on the interpretation of timing and pattern of metastatic dissemination.

## Cohort overview

**Fig. 1:**
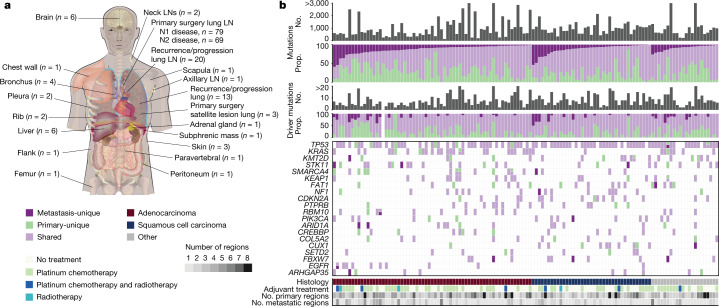
Sample distribution and mutational overview in the paired primary metastasis TRACERx 421 cohort. **a**, The distribution of metastatic samples by anatomical location; n indicates number of samples used in analyses. **b**, The total number of mutations and putative driver mutations detected per case (grey bars) and the proportion of these mutations that are unique to the primary tumour (green) or metastasis (dark purple), or shared between primary and metastasis (light purple) per case. The top 20 most frequently mutated cancer genes and their presence/absence in the primary and metastatic samples, including instances of two driver mutations in the same gene, are also shown. The histology, number of primary and metastatic samples sequenced, and adjuvant therapy status is illustrated. No., number; prop., proportion; LN, lymph node.

**Extended Data Fig. 1:**
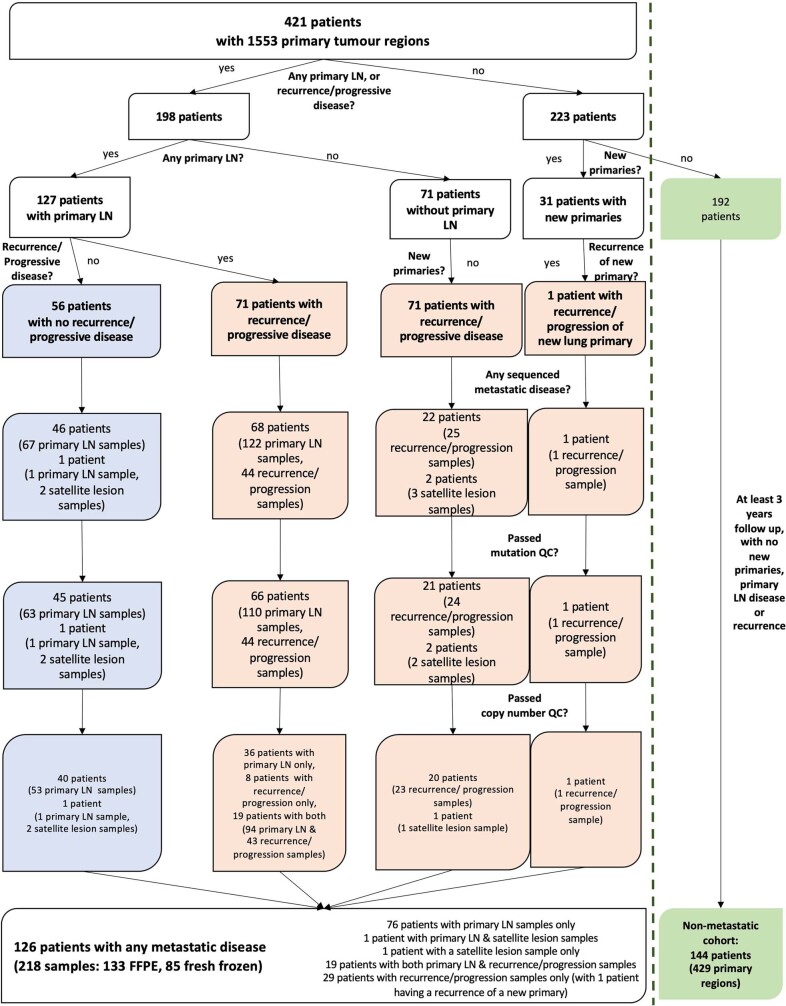
Cohort and sample overview. Sample acquisition and quality control overview, also highlighting the non-metastatic cohort. In addition to the 31 patients with new primary tumours highlighted in the figure, there are 10 other new primary cases within the metastatic cohort, totalling 41 new primary cases; LN, lymph node; QC, quality control; FFPE, formalin-fixed paraffin embedded tissue.

In the TRACERx 421 cohort, which encompasses 421 prospectively recruited patients with operable early-stage untreated NSCLC, 30.2% of patients (127 out of 421) were identified to have lymph node (LN) metastases at primary tumour surgical resection (N1/N2 disease). Primary LN samples (148 regions) from 96 patients were successfully sequenced and passed quality control checks (Fig. 1a and Extended Data Fig. 1). Three metastatic satellite regions from the primary surgery timepoint in two patients were also sequenced (Fig. 1a and Extended Data Fig. 1). Hereafter, we refer to primary LN metastases (148 regions) and satellite lesions (3 regions) resected at the time of surgery as ‘primary LN/satellite lesions’.

After a median follow up of 4.66 years (1,702 days; 95% confidence interval (CI) = 1,649–1,784 days), 33.7% (142 out of 421) of patients developed recurrent disease (median time to recurrence = 353.5 days; interquartile range (IQR) = 200–676.5 days). Recurrence/progression samples could not be obtained from 95 out of 142 patients owing to difficulty in accessing the site of disease (for example, the brain), patient frailty, patient preference or tumour samples failing quality-control criteria. An additional recurrence sample (one region) from a new primary lung cancer in one patient was also sequenced and included. A total of 67 recurrence/progression samples in 48 patients were successfully sequenced and passed quality control checks (Fig. 1a and Extended Data Fig. 1). There was an overlap of 19 patients with both primary LN/satellite lesions and subsequent recurrence/progression metastases. When performing analyses combining all metastatic sample types (primary LN, satellite, recurrence/progression samples), we refer to these as ‘metastases’. Hereafter, we refer to a ‘case’ as a primary tumour and its paired metastases.

In total, the WES data of 476 primary tumour regions paired with 218 metastatic primary LN/satellite and/or recurrence tumour samples in 126 patients passed quality control checks (Extended Data Fig. 1; median depth = 398×, IQR = 356–437; Methods). Detailed clinical features of patients are provided in Extended Data Table 1. A total of 144 patients within the TRACERx 421 cohort (429 primary tumour regions) who did not develop any primary LN disease, subsequent recurrence/progression, or any new primary tumours, and who had at least 3 years of follow up (median = 1,764 days, IQR = 1,523–1,854 days; Extended Data Fig. 1) were used as a control group for non-metastatic disease.

**Extended Data Fig. 2:**
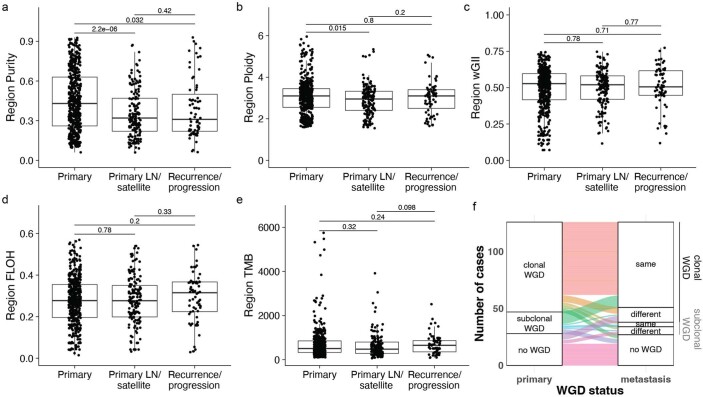
Genomic analyses of primary and metastases. **a**. Comparison of primary tumour and metastasis purity (median purity: primary = 0.43, primary LN/satellite = 0.32, recurrence/progression = 0.31, Wilcoxon rank-sum test), **b**. Comparison of primary tumour and metastasis ploidy (median ploidy: primary = 3.1, primary LN/satellite = 2.95, recurrence/progression = 3.1, Wilcoxon rank-sum test) **c**. Comparison of primary tumour and metastasis weighted genomic instability index (wGII, median wGII: primary = 0.53, primary LN/satellite = 0.52, recurrence/progression = 0.51, Wilcoxon rank-sum test) **d**. Comparison of primary tumour and metastasis fraction of the genome subject to loss of heterozygosity (FLOH, median FLOH: primary = 0.28, primary LN/satellite = 0.28, recurrence/progression = 0.32, Wilcoxon rank-sum test) **e**. Comparison of primary tumour and metastasis tumour mutation burden (TMB, median TMB: primary = 508, primary LN/satellite = 479, recurrence/progression = 651, Wilcoxon rank-sum test). **f**. Comparison of whole genome doubling (WGD) status between primary tumours and paired metastases. There is no enrichment in WGD in metastases (Fisher’s exact test, *p* = 1). The box plots represent the upper and lower quartiles (box limits), the median (centre line) and the vertical bars span the 5th to 95th percentiles. All tests were two-sided unless otherwise specified.

A comparison of matched primary tumours and metastases revealed a significantly lower tumour purity within metastases (median values = 0.43, 0.32 and 0.31 for primary, primary LN/satellite lesions and recurrence/progression samples, respectively; Wilcoxon rank-sum test, *P* = 2.2 × 10^−6^ and 0.032; Extended Data Fig. 2a). Although the primary LN/satellite lesions had a lower ploidy compared with the primary regions, this difference was small (median values = 3.1, 2.95 and 3.1 for primary, primary LN/satellite lesions and recurrence/progression samples, respectively; Wilcoxon rank-sum test, *P* = 0.015; Extended Data Fig. 2b). No significant difference was observed in whole-genome doubling (WGD) status, genome complexity (as measured by the weighted genomic instability index), fraction of the genome subject to loss of heterozygosity (FLOH) and tumour mutation burden (Extended Data Fig. 2c–f).

Metastasis-unique mutations, either not sampled or not detectable in the primary tumour, were identified in every case, including metastasis-unique driver mutations in 33.3% of cases (42 out of 126 cases; median number of metastasis-unique drivers per case = 0, IQR = 0–1; Fig. 1b and Extended Data Table 2). For example, an inactivating mutation in *STK11* (p.D194N) was identified exclusively in the primary LN metastasis of patient CRUK0691; and an activating mutation in *PIK3CA* (p.E545K) was identified in a primary LN metastasis of patient CRUK0451 and not in the primary tumour. However, the majority of driver mutations (68.6%) were shared between the primary and paired metastases (median number of shared drivers per case = 5, IQR = 3–7; Fig. 1b). Mutations in drivers such as *NRAS* and *RB1*, as well as *EGFR* exon19 deletions and L858R mutations, were always shared. By contrast, for *KRAS*, both shared and primary-unique activating mutations were identified (Fig. 1b), indicating the potential relevance of testing both the primary and metastatic sites for *KRAS* allele-specific targeted therapy stratification.

## Timing metastatic divergence

**Fig. 2:**
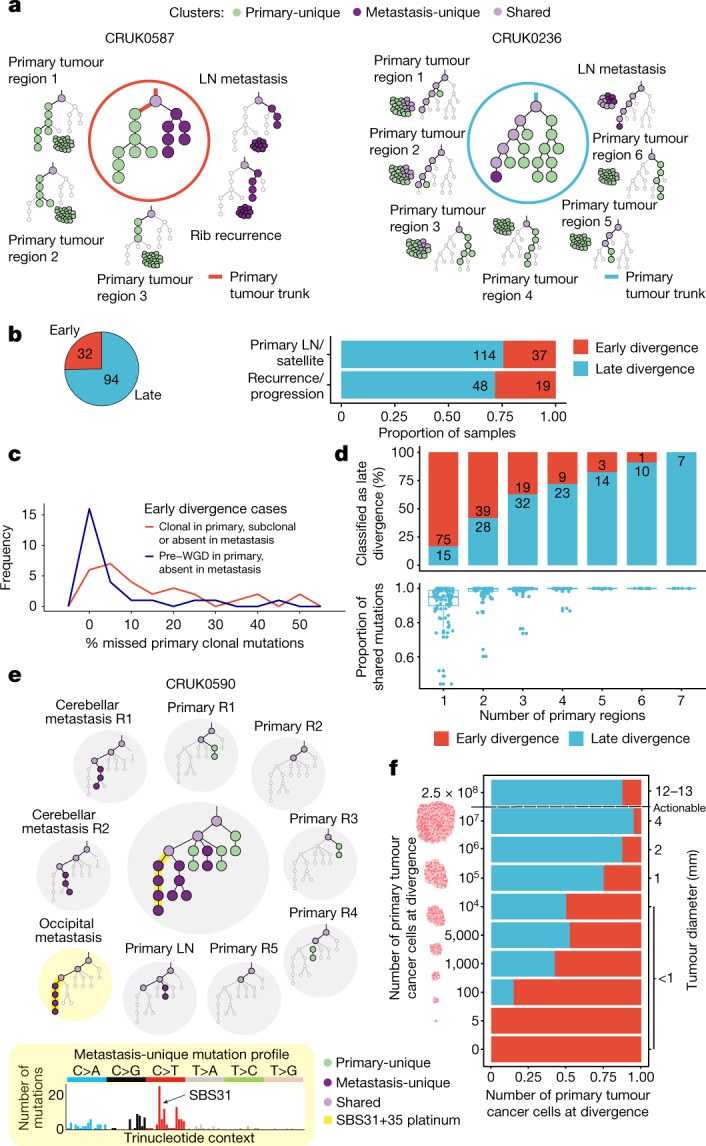
Timing metastatic divergence. **a**, Example phylogenetic trees depicting early (CRUK0587) and late (CRUK0236) divergence. Light purple, shared; dark purple, metastasis-unique; green, primary-unique mutation clusters. **b**, Pie chart showing the fraction of cases with early (*n *= 32) and late divergence (*n* = 94) (left panel). The proportion of early and late divergence by metastasis type at the sample level (primary LN/satellite versus recurrence/progression; Fisher’s exact test, *P* = 0.61)  (right panel). **c**, In early divergence cases, the median number of pre-WGD mutations (blue line) defined as not clonal in the metastases is 1.4% (IQR = 0.8–3.2%; *n* = 28). Post-WGD mutations or mutations in non-WGD tumours (red line) were more likely to be not clonal in the metastasis (median = 8.5%, IQR = 3.0–22.3%; *n* = 32; Wilcoxon rank-sum test, *P* = 0.003). **d**, Downsampling of late divergence cases (n = 94). A random set of primary tumour regions was used to re-classify the timing of divergence for each case (top panel); proportion of shared mutations between downsampled primary tumour and metastases (bottom panel). **e**, Phylogenetic trees for case CRUK0590 (inner circle) and within each region, depicting an active platinum signature in the occipital metastasis, timing metastatic divergence of the occipital and cerebellar metastases to a period when platinum therapy was delivered, approximately 6–8 months before recurrence. **f**, Simulations of tumour size (*n* = 20 simulations per tumour size) at metastatic clone divergence suggest that early divergence is more likely to happen when the primary tumour is small; a diameter ≥8 mm is a typical threshold used to investigate solid nodules detected using computed tomography^10–16^ (denoted ‘actionable’). The box plots represent the upper and lower quartiles (box limits), the median (centre line) and the vertical bars span the 5th to 95th percentiles. All tests were two-sided unless otherwise specified. R, region; LN, lymph node; WGD, whole genome doubling.

**Extended Data Fig. 3:**
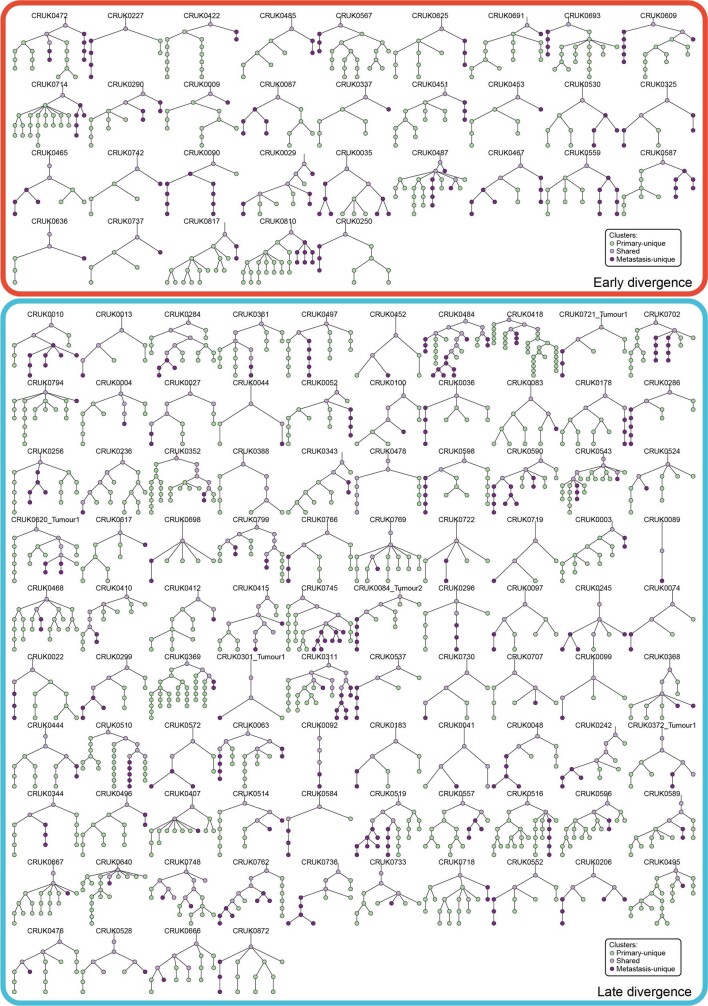
Overview of all phylogenetic trees. All phylogenetic trees for the 126 tumours with their paired metastases, split by timing of divergence (early vs. late). Clusters annotated in green are primary-unique, clusters in light purple are shared, while clusters in dark purple are metastasis-unique.

**Extended Data Fig. 4:**
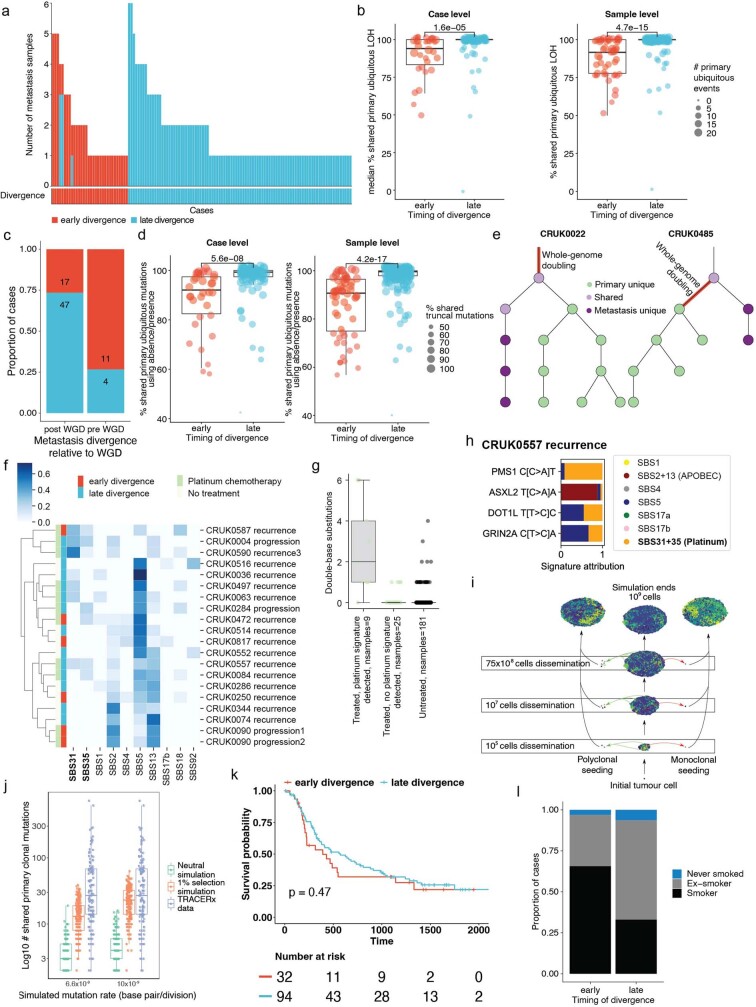
Timing of metastatic divergence. **a**. Sample level divergence timing (early and late). Where both early and late divergence is seen in multiple metastasis samples of one case, the overall timing is defined as early. **b**. Orthogonal method to time metastatic divergence using primary ubiquitous arm-level loss of heterozygosity (LOH). Arm level LOH was significantly more likely to be fully clonal in late compared to early divergence (case level median early = 0.94, late = 1, Wilcoxon rank-sum test, *p* = 1.6e-5; sample level median, early = 0.92, late = 1, Wilcoxon rank-sum test, *p* = 4.7e-15). **c**. Orthogonal method timing divergence using primary clonal whole genome doubling (WGD). There is enrichment of early divergence in pre-WGD divergence (Fisher’s exact test, *p* = 0.0017). **d**. Orthogonal method to time metastatic divergence using simple absence/presence of mutations in the primary tumour, to define primary ubiquitous mutations. Early divergent tumours have a lower proportion of shared primary ubiquitous mutations (case level median early = 92.1%, late = 99.3%, Wilcoxon rank-sum test, *p* = 5.6e-8; sample level median, early = 90.7%, late = 99.6%, Wilcoxon rank-sum test, *p* = 4.20e-17). **e**. Examples of pre- and post-WGD divergence (CRUK0485 and CRUK0022, respectively). The red line represents the branch with WGD. **f**. Detected mutational signatures using sample unique mutations for each of the metastatic samples with sufficient mutations (more than 50). SBS31 and SBS35 represent the platinum mutation signatures. **g**. In patients treated with platinum chemotherapy and where platinum signature was detected in the metastases (9 samples), an enrichment was seen in sample-specific double base substitutions (Mann-Whitney-U test; treated and detected platinum signature vs. treated and no signature detected (25 samples), *p* = 2.58e-5; treated and detected platinum signature vs. untreated (181 samples), *p* = 1.32e-10). **h**. In cases where platinum signature was detected, putative metastasis-unique driver mutations were mapped to the most likely signature. Example case of CRUK0557 where mapping such mutations (*PMS1*, *ASXL2*, *DOT1L*, *GRIN2A*) revealed *PMS1* to likely be platinum-driven. **i**. Schematic representation of the agent-based modelling approach used to investigate timing and patterns of metastatic seeding. **j**. Number of shared primary clonal mutations between simulated primary-metastasis pairs and the different mutations and selection rates. Additionally, the number of shared primary clonal mutations from TRACERx data is indicated. **k**. Kaplan-Meier analysis demonstrating no significant difference in early vs. late divergence (Log rank test, *p* = 0.47). **l**. Early divergence is associated with a higher proportion of current smokers (n early = 32, n late = 94; Fisher’s exact test, *p* = 0.005). The box plots represent the upper and lower quartiles (box limits), the median (centre line) and the vertical bars span the 5th to 95th percentiles. All tests were two-sided unless otherwise specified.

Phylogenetic trees were constructed for each case using our tool CONIPHER^4^, and the timing of metastatic divergence was estimated (defined as when the metastatic clone first existed, rather than when the cells migrated from the primary tumour; Methods). We defined two broad categories of metastatic divergence timing: early or late (Fig. 2a and Extended Data Fig. 3). For example, for patient CRUK0587, diagnosed with an adenosquamous carcinoma, with a sequenced primary LN metastasis and rib recurrence/progression sample, we identified a set of mutations that were clonal within all primary tumour regions yet entirely absent from the metastatic samples (Fig. 2a). This suggests that a complete clonal sweep occurred within the primary tumour after metastatic divergence. We designated such cases as early divergence. Conversely, for patient CRUK0236, diagnosed with a lung squamous cell carcinoma (LUSC), the clonal mutations present in all primary tumour regions were also present in every cancer cell of the sequenced primary LN metastasis. In this case, after metastatic divergence, there were no additional clonal sweeps within the primary tumour and divergence could be classified as late. Overall, 74.6% (94 out of 126) of cases exhibited late divergence, whereas 25.4% (32 out of 126) exhibited early divergence (Fig. 2b and Extended Data Fig. 4a). For cases with multiple metastatic samples that displayed a mix of early and late divergence, the overall timing at the case level was designated as early (Extended Data Fig. 4a). The proportions of early versus late divergence were similar in primary LN/satellite lesions and subsequent recurrence/progression metastases (Fisher’s exact test, *P* = 0.61; Fig. 2b).

Orthogonal methods to time divergence, using loss of heterozygosity (LOH; a ratchet-like irreversible process during cancer evolution), primary clonal WGD and the proportion of primary-ubiquitous mutations present in the metastases support the findings that metastases usually diverge late (Methods and Extended Data Fig. 4b–d). Even in cases of early divergence, the majority of primary-ubiquitous mutations (median across cases = 92.1%; IQR = 82.5–97.4%) were shared between the metastases and their paired primary tumours, suggesting that early divergence probably occurs relatively late in molecular evolution time (Extended Data Fig. 4d).

WGD in the primary tumour can be used to provide further granularity to the timing of metastatic divergence. Clonal primary WGD was detected in 79 out of 126 primary tumours. Metastatic divergence most often occurred after primary clonal WGD (64 out of 79; 81.0%). In a minority of WGD cases (11 out of 79; 13.9%), metastatic divergence occurred both before a clonal sweep in the primary tumour and before the WGD event (Extended Data Fig. 4c,e). In these 11 cases, a median of 9.7% (IQR = 5.8–21.3%) of primary-ubiquitous mutations were absent in metastases, highlighting that both metastatic divergence and WGD were nevertheless late in molecular evolutionary time. Notably, in 6 out of 11 of the pre-WGD early divergence cases, a parallel subsequent WGD event took place in the metastasis. Overall, mutations occurring pre-WGD were significantly less likely to be not clonal in the metastases compared with other primary-ubiquitous mutations (median percentage of not clonal pre-WGD mutations = 1.4%, IQR = 0.8–3.2%; median percentage of not clonal post-WGD or non-WGD mutations = 8.5%, IQR = 3.0–22.3%; Wilcoxon rank-sum test, *P* = 0.003; Fig. 2c), indicating that pre-WGD mutations might make better therapeutic targets including in personalized immune-based therapies.

The impact of primary tumour sampling on timing metastatic divergence was also investigated. This timing is dependent on correctly classifying mutation clonality within the primary tumour. Undersampling of the primary tumour may result in an illusion of clonality, whereby subclonal mutations are erroneously inferred as clonal within a single region^5^. Indeed, when using only a single randomly down-sampled primary tumour region to define the timing of divergence, 75 out of 90 (83.3%) late divergence cases were incorrectly classified as early (Fig. 2d).

To evaluate whether the platinum mutational signature could be used to further time the divergence of recurrence/progression samples, we examined the mutational signatures in the recurrence/progression samples^6–8^. Out of the 67 recurrence/progression samples from 48 patients (26 of whom were treated with adjuvant platinum therapy), 20 recurrence/progression samples from 19 patients had sufficient metastasis-unique mutations to examine the underlying mutational signatures. Ten of these patients were treated with adjuvant platinum therapy and nine patients were not. The platinum mutational signature was identified in the majority of these treated recurrence/progression samples (9 out of 11; 81.8%), with 7 out of 9 samples being classified as late divergence (Extended Data Fig. 4f). Orthogonal validation revealed a significantly higher proportion of metastatic sample-specific double-base substitutions compared with the 181 metastatic samples from patients who did not receive platinum therapy (Mann–Whitney *U*-test, *P* = 1.32 × 10^−10^, Extended Data Fig. 4g). We identified one case in which two closely related brain metastases, identified at first recurrence, appeared to diverge from their common ancestor during or after adjuvant platinum chemotherapy, which was given 6–8 months before recurrence and resection of both brain metastases (CRUK0590; Fig. 2e). This was evidenced by the presence of platinum-associated mutations in the occipital metastasis, but not in the cerebellar metastasis. In another case, CRUK0557, we identified a metastasis-unique putative driver mutation in *PMS1* that occurred in a platinum-signature trinucleotide context (Extended Data Fig. 4h).

Finally, we used a modified version of the in silico spatially explicit model from Sun et al.^9^ to simulate the growth of a tumour (Methods). The evolution of individual cells was tracked under differing, biologically informed mutation rates and dynamic selection pressures to generate simulated bulk primary tumours and paired metastases that diverged at known, prespecified primary tumour sizes. The proportion of early and late metastatic clone divergence was then estimated (Methods and Extended Data Fig. 4i–j). The results demonstrate an increasing proportion of early metastatic divergence with reducing tumour size (Fig. 2f). When the primary tumour consisted of 2.5 × 10^8^ cancer cells (which equates to a tumour diameter of 12–13 mm, assuming a tumour purity of 37%, the median in our cohort), 14% of simulations were classified as early (86% late). By contrast, for simulations with divergence below 1 mm diameter, 78% of divergence was classified as early (22% late). Thus, in early divergence cases (32 out of 126 of sequenced metastatic TRACERx cases), the simulations suggest that metastatic divergence is more likely to occur when the tumour diameter is less than 8 mm, which is the typical size threshold used to guide further investigations in modern solid nodule management protocols^10–16^, potentially limiting the use of computed tomography screening in these tumours.

With the exception of smoking, we observed no significant associations between timing of metastatic divergence and lung cancer-specific disease-free survival or clinical characteristics (Fisher’s exact test, *P* = 0.005; Extended Data Fig. 4k,l and Extended Data Table 3). Smoking status at the time of primary tumour resection remained an independent predictor of early divergence in logistic regression analyses accounting for patient age, stage, histology and adjuvant treatment (generalized linear model using binomial distribution; ANOVA *χ*^2^, *P* = 0.016).

## Modes of dissemination

**Fig. 3:**
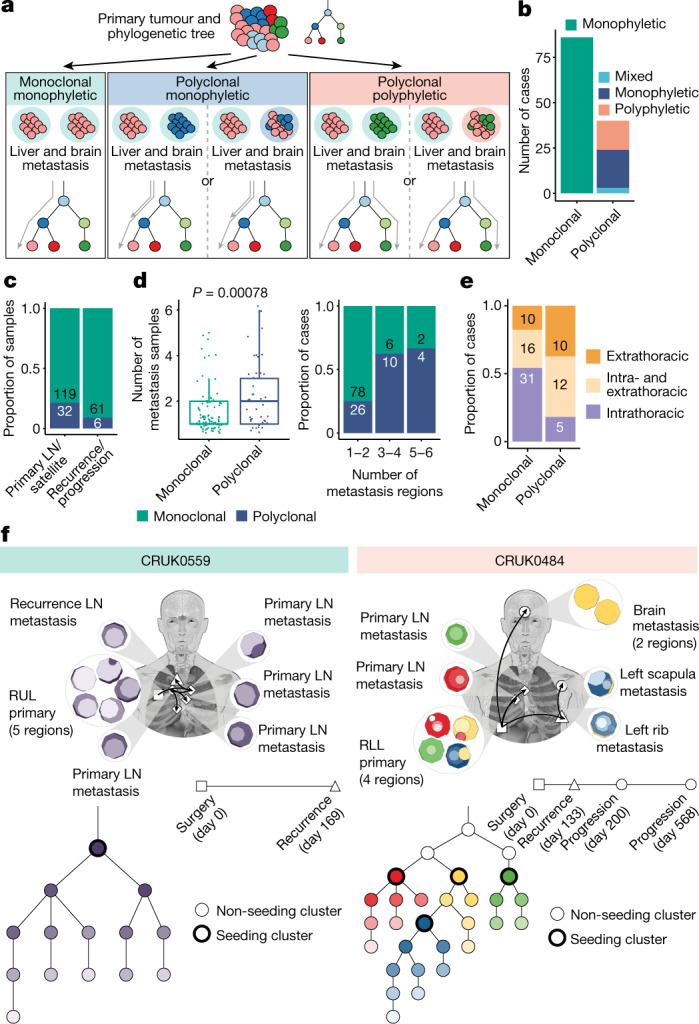
Modes of dissemination. **a**, Definitions of the dissemination patterns of metastases at the case level, described relative to the primary tumour phylogeny. Grey arrows indicate the branches leading up to the seeding cluster(s). **b**, The most prevalent mode of metastatic dissemination observed is monoclonal monophyletic. Polyclonal ‘mixed’ represents cases in which a consensus dissemination pattern could not be inferred due to different possible phylogenetic tree topologies. **c**, At the sample level, polyclonal dissemination is more prevalent in primary LN/satellite lesions compared to recurrence/progression lesions (Fisher’s exact test, *P* = 0.03). **d**, Polyclonal dissemination is associated with a higher number of metastatic samples compared with monoclonal dissemination (median number of metastasis samples: 2 versus 1, respectively; Wilcoxon rank-sum test, *P* = 0.00078), sample number depicted as discrete values (left panel) or proportion (right panel). **e**, In cases where recurrence occurs, polyclonal dissemination is associated with extrathoracic metastasis, as identified on imaging, compared with monoclonal dissemination (*n* = 57 (monoclonal), *n* = 27 (polyclonal); Fisher’s exact test, *P* = 0.0056) **f**, Examples of cases with monoclonal (CRUK0559) and polyclonal polyphyletic (CRUK0484) dissemination patterns, both of which also demonstrate metastases being seeded from other sites of metastatic disease. The black arrows on the body map represent the routes of metastatic seeding (MACHINA). Each seeding cluster in the phylogenetic tree, as defined by our method, is assigned a unique colour that is also represented in the region clone maps. The timeline indicates the day on which the metastases were detected on imaging; the biopsy dates differ from this. For CRUK0559, the recurrence biopsy took place on day 188. For CRUK0484, the rib recurrence, scapula progression and brain progression were sampled at days 147, 433 and 582, respectively. The box plots represent the upper and lower quartiles (box limits), the median (centre line) and the vertical bars span the 5th to 95th percentiles. All tests were two-sided unless otherwise specified. LN, lymph node; RUL, right upper lobe; RLL, right lower lobe.

**Extended Data Fig. 5:**
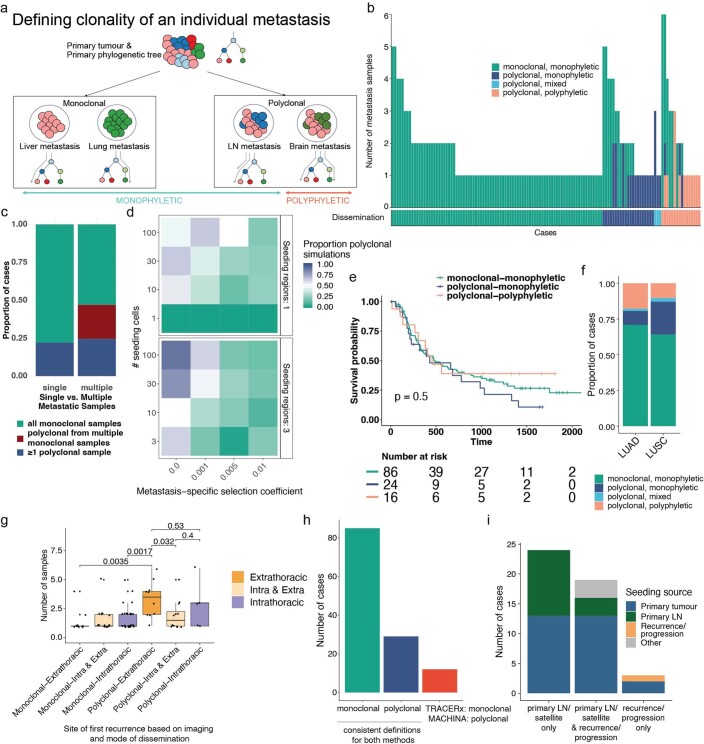
Modes of dissemination. **a**. Sample level definitions of dissemination patterns relative to the primary tumour phylogeny. **b**. Sample level dissemination patterns with overall case level defined beneath **c**. Proportion of cases defined as polyclonal or monoclonal divided by whether a single or multiple metastatic samples were available (n single = 77, n multiple = 49). There is increased power to detect polyclonal seeding when multiple metastatic samples were sequenced (in dark red, we see approximately 22.4% of polyclonal cases result from multiple monoclonal seeding patterns). **d**. Proportion of observed polyclonal metastases when simulating differing numbers of disseminating primary tumour cells (y-axis) and varying the number of primary regions from which this occurs (top and bottom panel). The primary tumour was always simulated with 1% selection while the selection coefficients were varied in the metastasis (x-axis). Increasing selection pressure in the metastasis is associated with the appearance of monoclonal dissemination even if the dissemination from the primary tumour is polyclonal. The fewer the number of disseminating cells, the stronger the effect. **e**. Kaplan-Meier analyses demonstrate no significant difference in lung-cancer specific disease-free survival across the different dissemination patterns (Log rank test, *p* = 0.5). **f**. Proportion of dissemination type on a case level, as seen in the main histologic subtypes (LUAD, n = 65; LUSC, n = 39; Fisher’s exact test, *p* > 0.05). **g**. Tumours with polyclonal dissemination and extrathoracic metastases have more metastatic samples acquired (Wilcoxon rank-sum test). **h**. Comparison of the TRACERx dissemination definitions with MACHINA^18^ shows that the majority of dissemination patterns are consistent across the two methods, with only 12/126 cases differing; with the TRACERx definitions being more conservative by classifying these cases as monoclonal whereas MACHINA defines these as polyclonal. **i**. Summary of MACHINA analysis of a metastasis seeding other sites of disease in 46 cases with multiple metastatic samples. ‘Other’ represents cases where the primary tumour seeds the recurrence and additional metastasis seeding patterns are concurrently observed (e.g., recurrence/progression sample seeding the primary LN, primary LN to primary LN seeding, recurrence seeding a progression sample). The box plots represent the upper and lower quartiles (box limits), the median (centre line) and the vertical bars span the 5th to 95th percentiles. All tests were two-sided unless otherwise specified; LN, lymph node.

To gain further insights into patterns and anatomical sites of metastatic dissemination and whether this involved a single subclone (monoclonal) or multiple genetically distinct subclones (polyclonal) from the primary tumour, multi-region sampling and clonal architecture analysis together with clinical case report forms and imaging analyses were used. Both our tree-building and clonal architecture methods were extensively benchmarked to ensure the validity of the results^4,17^. In the following analysis we refer to metastatic monoclonal and polyclonal dissemination relative to the primary tumour, across all sampled metastases within an individual case (Fig. 3a and Methods). This contrasts with an approach by which clonality of dissemination is defined relative to an individual metastasis sample (Extended Data Fig. 5a). We further explored whether polyclonal dissemination stemmed from a single or multiple branches of the evolutionary tree, reflecting monophyletic or polyphyletic dissemination, respectively.

In 31.7% (40 out of 126) of cases, we observed polyclonal dissemination, whereby multiple primary tumour clones seeded metastases (Fig. 3b). Of the 40 metastases with polyclonal dissemination, 21 were monophyletic and 16 were polyphyletic (Fig. 3b and Extended Data Fig. 5b); by contrast, for 3 tumours, both dissemination patterns were compatible with multiple possible phylogenetic tree topologies (Fig. 3b and Extended Data Fig. 5b). In the remaining 68.3% (86 out of 126) of cases, monoclonal dissemination was identified (Fig. 3b). Polyclonal dissemination was enriched in primary LN/satellite lesions compared to recurrence/progression samples (Fisher’s exact test, *P* = 0.03; Fig. 3c).

The number of metastatic samples sequenced was significantly higher in cases with inferred polyclonal dissemination compared with monoclonal dissemination (Wilcoxon rank-sum test, *P* = 0.00078; Fig. 3d). Furthermore, in 11 cases, we observed evidence for each individual metastatic site demonstrating monoclonal dissemination, yet at the case level, the multiple sampled metastases originated from multiple distinct seeding clones within the primary tumour, rendering the case-level inference as polyclonal dissemination (Extended Data Fig. 5b,c). These data suggest that undersampling of metastases can lead to dissemination pattern mischaracterization. Whereas polyclonal dissemination is almost always accurate, monoclonal dissemination may reflect a mixture of true monoclonal dissemination and undetected polyclonal dissemination. Thus, the extent of polyclonal dissemination reported here is probably an underestimate.

In 16.3% (14 out of 86) of cases with monoclonal dissemination, we observed solely subclonal and not clonal metastasis-unique mutations in some the paired metastatic samples, suggesting that there were no additional clonal sweeps at these metastatic sites. In these cases, the majority of which exhibited late divergence (12 out of 14), the timing of metastatic divergence may be equivalent to the timing of metastatic dissemination. In the remaining cases with metastasis-unique clonal mutations (72 out of 86), either the clone that seeded the metastasis was not sampled within the primary tumour or, after dissemination, additional clonal sweeps occurred, indicating ongoing selection within the metastasis (Extended Data Fig. 5d).

With the exception of location of disease recurrence, there was no significant association between dissemination pattern and lung cancer-specific disease-free survival nor histological/patient clinical characteristics (Extended Data Fig. 5e,f and Extended Data Table 3). Even after controlling for a higher number of metastases sampled, polyclonal dissemination (at the case level, from both primary LN/satellite lesions and recurrence/progression samples) was enriched for tumours that result in extrathoracic recurrence compared with monoclonal dissemination (Fisher’s exact test, *P* = 0.0056 (Fig. 3e); linear modelling adjusting for metastases sampled, *P* = 0.006 (Extended Data Fig. 5g)).

Finally, we used MACHINA^18^ as an orthogonal assessment of dissemination patterns, revealing 90% result concordance with our method (Methods, Extended Data Fig. 5h and Supplementary Note). We also examined migration histories and evaluated the likelihood of new metastatic sites being seeded and colonized by cancer cells from other metastases rather than the primary tumour using MACHINA. Although the identification of different seeding patterns may be limited by the number of distinct metastases sequenced per patient, metastatic sites were identified as likely seeded from other metastases in 38% (18 out of 47) of cases from whom multiple metastatic samples were available (for example, CRUK0559, Fig. 3f and Extended Data Fig. 5i). To explore whether primary LN disease acts as a gateway for further metastasis, we focused our analysis on the 19 cases that had both primary LN metastases and subsequent recurrence/progression samples. In 13 out of 19 cases, we found that dissemination probably occurred solely from the primary tumour. In the remaining six cases, we identified three cases in which the primary LN metastases seeded the subsequent recurrences, and three cases in which the recurrence/progression samples, rather than the primary LN, seeded other metastases. An example of the latter pattern is a case of polyclonal polyphyletic dissemination (CRUK0484, pleomorphic carcinoma; Fig. 3f), where we found evidence for four distinct subclones in the primary tumour separately seeding two primary LN metastases, a rib metastasis (day 133) and a subsequent brain metastasis (day 568). In this case, MACHINA predicted that the initial clinically detected rib metastasis seeded the subsequent scapular metastasis (day 200).

## Selection in metastases

To investigate whether certain genomic events in the primary tumour conferred metastatic potential, the seeding clone(s) for each metastasis was identified and its genomic features explored and compared to non-seeding clones within the same tumour. We focused our analysis on mutations specific to the seeding clone (referred to as the seeding cluster). In total, we identified 196 seeding clusters in the 126 cases, of which 50 seeding clusters were truncal (25.5%). Notably, the seeding cluster represents mutations found in primary tumours that predate any exposure to adjuvant chemotherapy or radiotherapy. The remaining non-seeding clusters were classified as either ‘shared’ if present in both the primary tumour and metastasis, or ‘primary-unique’ or ‘metastasis-unique’.

**Fig. 4:**
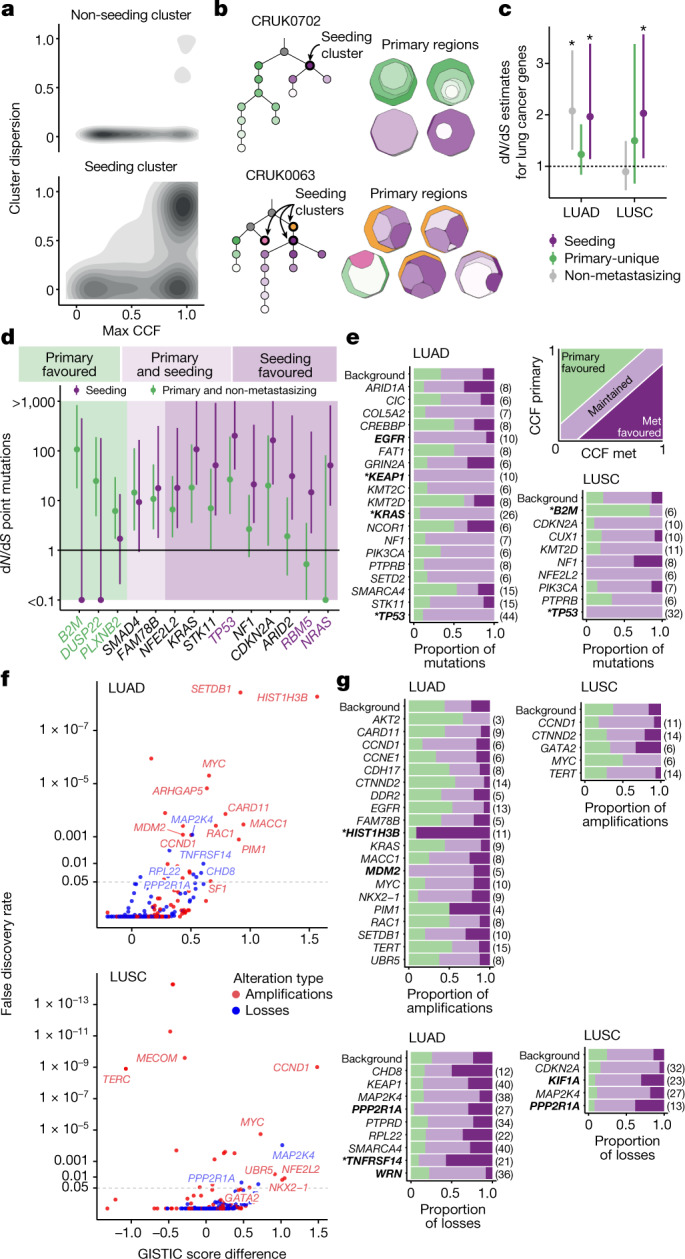
Selection in metastasis. **a**, Cluster dispersion and maximum cancer cell fraction (CCF) across primary tumour regions in the subclonal seeding clusters versus non-seeding clusters in metastasizing tumours. **b**, Examples of seeding cluster dispersion across primary tumour regions illustrated by one clone-map per region. CRUK0702 demonstrates a single dominant seeding cluster (purple), dispersed across two primary tumour regions. CRUK0063 highlights two dominant (purple and yellow) and one minor seeding cluster (pink). **c**, Cohort-level selection (*n* = 111 genes) of seeding (purple) versus primary-unique mutations from metastasizing tumours (green) versus subclonal non-metastasizing primary tumour mutations (grey). The dots represent d*N*/d*S* estimates; the asterisks indicate values that are significantly different from 1. **d**, Gene-level d*N*/d*S* values of seeding mutations versus combined primary-unique/non-metastasizing primary tumour mutations for all histologies. A d*N*/d*S* odds ratio (OR) of >2 indicates a seeding favoured gene; <0.5 is primary favoured; 0.5–2 is classified as both primary and seeding favoured. Purple and green gene names represent significant enrichment in seeding and non-seeding mutations, respectively. The lines indicate the 95% CIs. **e**, Paired primary tumour–metastasis (met) mutation analysis. Metastasis favoured mutations are defined as having a higher clonality in metastases compared with the primary tumour; primary favoured if the clonality is higher in the primary tumour; the remaining were classified as maintained; background refers to mutations in non-cancer genes. **f**, The GISTIC2.0 score difference between the unpaired metastases and non-metastasizing cohorts plotted against the false-discovery rate of the *G*-score in the metastases cohort for cancer genes. Amplified genes are shown in red; deleted genes are shown in blue. Horizontal dotted lines indicate *p* = 0.05 **g**, Paired SCNA analysis of cancer genes that were found to be significant in **f**. An amplification/deletion was classified as metastasis favoured if it was present in the metastasis and absent in the primary tumour, primary favoured if present in the primary tumour but not the metastasis, or otherwise defined as maintained. Only tumours that had at least one copy number event in the gene in any sample were counted. For **e** and **g**, significant genes (multinomial test; *p* < 0.05) are shown in bold; asterisks represent significance after multiple-testing correction (*q* < 0.05); numbers in parentheses indicate number of events.

**Extended Data Fig. 6:**
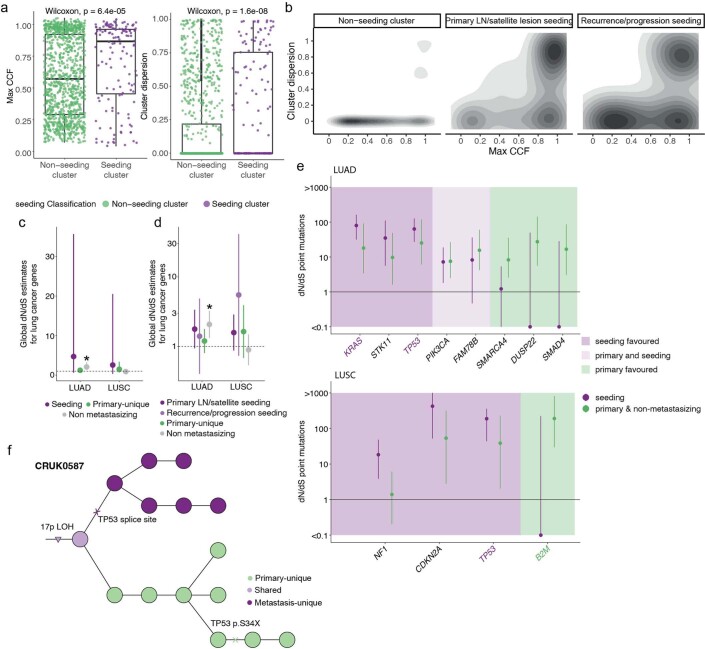
Mutation selection in metastases. **a**. Comparison of maximum cancer cell fraction (CCF) in subclonal primary-unique and seeding clusters (Wilcoxon rank-sum test, *p* = 6.4e-5) and clonal dispersion of primary-unique and seeding clusters (Wilcoxon rank-sum test, *p* = 1.6e-8). **b**. Higher dispersion and CCF is seen in the seeding clusters of both primary LN/satellite lesions and recurrence/progression samples compared to non-seeding clusters. Clusters that are found in both primary LN/satellite lesions and recurrence/progression samples were excluded from this analysis. **c**. Cohort level selection (n genes = 111) of only subclonal mutations in seeding vs. primary-unique mutations vs. mutations in non-metastasizing primary tumours. **d**. Cohort level selection (n genes = 111) of primary LN/satellite lesions vs. recurrence/progression seeding mutations vs. primary-unique mutations vs. mutations in non-metastasizing primary tumours. Dots represent dN/dS estimates; the asterisks indicate values that are significantly different from 1. **e**. Gene-level dN/dS values of seeding mutations vs primary-unique and non-metastasizing primary tumour mutations split by lung adenocarcinoma (LUAD) and lung squamous cell carcinoma (LUSC). Genes are classified as seeding favoured if the odds ratio (OR) of dN/dS of seeding vs. primary-unique mutations >2, primary favoured if OR <0.5, and otherwise classified as both primary and seeding favoured. Genes highlighted in purple and green are significantly enriched in seeding and non-seeding mutations respectively. **f**. Phylogenetic tree of CRUK0587. Clusters annotated in green are primary-unique, clusters in light purple are shared, while clusters in dark purple are metastasis-unique. There is a metastasis-unique *TP53* splice site mutation which occurred independently of a primary-unique S34X *TP53* mutation. Lines indicate the 95% confidence intervals for **c**,**d** and **e**. The box plots represent the upper and lower quartiles (box limits), the median (centre line) and the vertical bars span the 5th to 95th percentiles. All tests are two-sided unless otherwise specified.

In the accompanying Article, we found that patients whose tumours contained a recent large subclonal expansion in at least one primary tumour region had reduced disease-free survival^17^. We therefore examined the differences in the size of expansions between seeding and non-seeding clusters in the primary tumour and whether this reflected selection. Although seeding clusters can be truncal, to avoid biassing the results, we restricted the analysis to a comparison of subclonal seeding and non-seeding clusters. The maximum cancer cell fraction (CCF) across all regions of the primary tumour was significantly higher in seeding clusters than in non-seeding clusters (Wilcoxon rank-sum test, *P* = 6.4 × 10^−5^; Fig. 4a and Extended Data Fig. 6a), and seeding clusters were more dispersed across primary tumour regions (Methods; Wilcoxon rank-sum test, *P* = 1.6 × 10^−8^; Fig. 4a,b and Extended Data Fig. 6a). Similar results were observed when separating primary LN/satellite lesions and recurrence/progression samples (Extended Data Fig. 6b). These results suggest that, at the time of surgical resection, clones with metastatic potential were more likely to have undergone a subclonal expansion within the primary tumour. A similar phenomenon was found in the accompanying Article using circulating tumour DNA to track metastatic disease^19^.

To evaluate whether the expansion of the seeding cluster reflects a fitness advantage, we applied the dNdScv method^20^ to a curated set of lung cancer genes^20,21^ (Methods). In both lung adenocarcinoma (LUAD) and LUSC, when considering all seeding clusters combined, we observed significant positive selection of lung cancer-specific genes (LUAD, d*N*/d*S* = 1.97, 95% CI = 1.14–3.38; LUSC, d*N*/d*S* = 2.03, 95% CI = 1.16–3.57; Fig. 4c). In LUAD, the subclonal mutations in non-metastasizing primary tumours also showed significant positive selection (seeding cluster, d*N*/d*S* = 1.97, 95% CI = 1.14–3.38; primary-unique clusters, d*N*/d*S* = 1.23, 95% CI = 0.84–1.82; non-metastasizing primaries, d*N*/d*S* = 2.08, 95% CI = 1.32–3.25; Fig. 4c). In LUSC tumours, primary-unique subclonal clusters showed no significant positive selection for cancer genes (d*N*/d*S* = 1.5, 95% CI = 0.66–3.38; Fig. 4c), consistent with a substantial fraction of the non-metastatic mutations reflecting neutral evolution. Furthermore, the subclonal mutations in primary non-metastasizing LUSC tumours showed no significant positive selection (d*N*/d*S* = 0.89, 95% CI = 0.53–1.49;Fig. 4c). To investigate whether these results were driven solely by truncal seeding clusters, we restricted our analysis to subclonal mutations and observed similar, yet non-significant, d*N*/d*S* values (Extended Data Fig. 6c). There was no difference in selection when separating the primary LN/satellite lesions and recurrence/progression samples (Extended Data Fig. 6d).

To evaluate whether specific genes were subject to selection in metastasizing clones, we performed a d*N*/d*S* analysis of mutations in seeding and non-seeding clusters individually. Although 9 genes exhibited higher d*N*/d*S* ratios for seeding cluster mutations compared with non-seeding cluster mutations, only three were significantly higher—*NRAS*, *RBM5* and *TP53* (Benjamini-Hochberg (BH) correction, *q* = 0.019, 0.019 and 5.92 × 10^−6^ respectively; Fig. 4d).

To further evaluate these cancer genes in the context of primary to metastatic transition, we performed a paired analysis of driver mutations. We classified each mutation as metastasis favoured if it was present at a higher CCF in any metastasis compared with in its matched primary tumour; maintained, if it was present equally in the primary tumour and metastasis; or primary favoured if it was absent or present at lower frequency in the metastasis (Fig. 4e and Methods). We next compared these proportions for mutations in cancer genes against the proportions in non-driver mutations (defined as ‘background’).

In LUAD, mutations in *KRAS*, *TP53, KEAP1* and *EGFR* were maintained significantly more than background mutations; however, after multiple testing correction, only *KRAS*, *TP53* and *KEAP1* remained significant (multinomial test with BH correction, *q* = 0.0009, *q* = 2.9 × 10^−5^ and *q *= 0.043, respectively; Fig. 4e). In LUSC, *TP53* mutations were also significantly maintained (multinomial test with BH correction, *q* = 8.4 × 10^−5^, Fig. 4e). Similar results for *TP53* were seen when comparing d*N*/d*S* estimates in seeding clusters and primary-unique clusters (d*N*/d*S* 187.84 versus 38.62 respectively, Fig. 4d). These data suggest that, in the context of metastasis, *TP53* mutations are almost always associated with metastatic seeding, consistent with positive selection in both the primary and seeding clones (Fig. 4e and Extended Data Fig. 6e). In one case (CRUK0587; adenosquamous carcinoma) we observed evidence of parallel subclonal inactivation of *TP53*—in addition to a clonal LOH event encompassing 17p, we observed a stopgain *TP53* driver mutation (S34X) present in one of the primary regions while a distinct splice site driver mutation was observed in the metastatic samples (Extended Data Fig. 6f). No cancer genes harboured a significant enrichment for metastasis favoured mutations in either histological subtype. In LUSC, mutations in *B2M* were significantly primary favoured compared with the background (multinomial test with BH correction, *q* = 0.027; Fig. 4e and Extended Data Fig. 6e), suggesting that antigen presentation disruption through *B2M* mutation is not significantly selected at metastatic transition in LUSC. No significant differences were observed in the distributions of driver mutations when comparing adjuvant-treated and non-adjuvant-treated recurrence/progression samples (*χ*^2^ test, *P* = 0.83), suggesting that there is no detectable impact on selection of mutations in cancer genes by the use of adjuvant therapy.

We next examined the somatic copy number alteration (SCNA) landscape of primary and metastatic tumours using both unpaired and paired analyses. First, for the unpaired analysis, we separately applied GISTIC2.0^22^ to obtain an SCNA positive-selection score (*G*-score) and significance level (*q* value) at each genomic location for non-metastatic primary tumours and metastases samples from metastasizing tumours. This enabled the identification of loci with more recurrently aberrant copy number states in a metastatic phenotype compared with non-metastatic primary tumours (*G*-score difference (GSD); Methods). In all of the subsequent analyses, we report the *q* value for the metastatic cohort. We next performed paired analyses by classifying SCNAs overlapping significant loci from the unpaired analysis into three categories relative to their matched primary tumour: primary favoured, metastasis favoured or maintained (that is, found both in the primary tumour and its paired metastasis). We tested the SCNA classifications in comparison to a background distribution of non-driver gene SCNA classifications (multinomial test; Methods).

**Extended Data Fig. 7:**
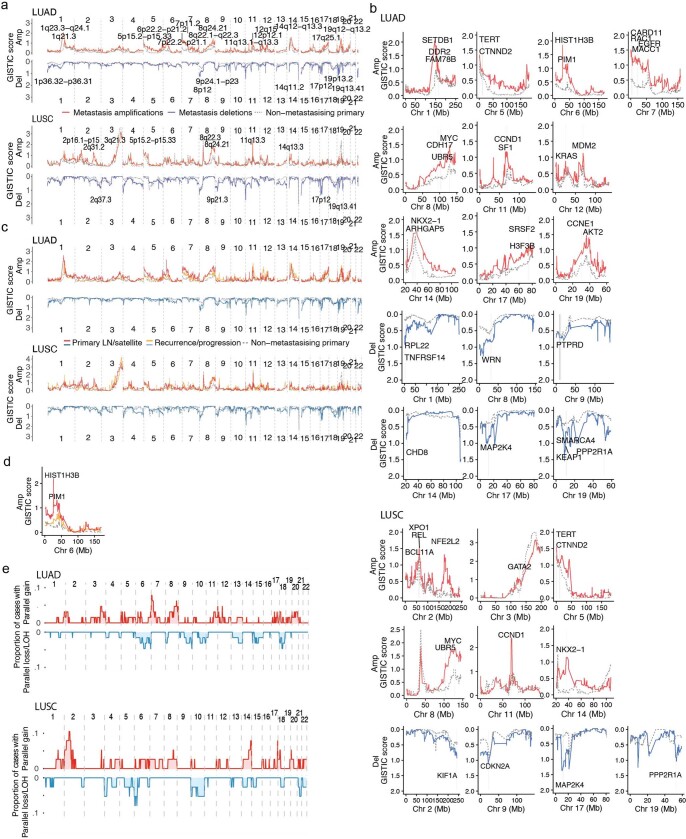
Somatic copy number aberration selection in metastases. **a**. Across-genome GISTIC2.0 scores are plotted for amplifications and deletions for lung adenocarcinoma (LUAD) and lung squamous cell carcinoma (LUSC). Annotated cytobands contain genes overlapping loci with significant G-scores in the metastasis cohort and that have a GISTIC2.0 score difference (GSD) >0 between unpaired metastases and non-metastasizing tumours. **b**. Individual chromosome plots highlighting genes overlapping significant loci in the metastasis cohort with GSD > 0 that were detected in the unpaired analysis performed in **a**. **c**. Across-genome GISTIC2.0 scores are plotted for amplifications and deletions for LUAD and LUSC separating primary LN/satellite lesions and recurrence/progression samples. **d**. Individual plot highlighting GSD between primary LN/satellite lesions, recurrence/progression samples and non-metastatic primary regions on chromosome 6 encompassing *HIST1H3B*. The locus is significantly amplified in the primary LN/satellite lesions (GSD = 1.90, *q* = 1.30e-7). **e**. Across genome plot showing the frequency of parallel gains/amplification events in red, and frequency of parallel loss/LOH events in blue. The top and bottom panels show the parallel evolution between primary regions harbouring the seeding clone and their paired metastases in LUAD and LUSC respectively; Amp, amplification; Del, deletion; Chr, chromosome; Mb, megabase.

In the unpaired analyses of LUSC metastases and non-metastasizing primary tumours, focal amplifications that were significantly recurrent in metastases with higher G-scores compared with non-metastatic primaries were identified in 11q13.3 (encompassing *CCND1*, GSD = 1.483, *q* = 9.72 × 10^−10^) and 2q31.2 (encompassing *NFE2L2*, GSD = 1.048, *q* = 0.0118; Fig. 4f and Extended Data Fig. 7a,b). In unpaired analyses of the LUAD cohort, focal amplifications identified as significantly recurrent in metastases with higher *G*-scores compared with non-metastatic primaries included 1q21.3 (encompassing *SETDB1*, GSD = 0.918, *q* = 3.70 × 10^−9^), 6p22.2 (encompassing *HIST1H3B*, GSD = 1.566, *q* = 5.31 × 10^−9^) and 12q15 (encompassing *MDM2*, GSD = 0.432, *q* = 8.34 × 10^−4^; Fig. 4f and Extended Data Fig. 7a,b). The latter two loci were significantly more metastasis favoured (*HIST1H3B*, multinomial test, *P* = 3.76 × 10^−6^) and maintained (*MDM2*, multinomial test, *P* = 0.0419; Fig. 4g), respectively, compared with the background in the paired analysis. Notably, in both unpaired LUAD and LUSC analyses, losses affecting 19q13.41 (encompassing *PPP2R1A*) were significantly recurrent in metastases (GSD = 0.5456, *q* = 0.0325; GSD = 0.6967, *q* = 0.0282, respectively); however, in the paired LUAD analyses, this loss was significantly metastasis favoured (multinomial test, *P* = 0.0122), whereas, in LUSC, it was significantly maintained (multinomial test, *P* = 0.0402; Fig. 4g). The results of the unpaired analyses were broadly consistent between primary LN/satellite lesions and recurrence/progression samples (Extended Data Fig. 7c), with the exception of amplification of *HIST1H3B* in LUAD, which was significant only in primary LN/satellite lesions and not in recurrence/progression samples (GSD = 1.902, *q* = 1.30 × 10^−7^; GSD = 0.301, *q* = 1, respectively; Extended Data Fig. 7d).

Furthermore, we observed parallel gains between distinct alleles of metastasizing primary tumour regions and their paired metastases in loci that were also found to be recurrently gained in metastases in unpaired LUAD analyses (Methods and Extended Data Fig. 7e). These loci included 7p22.3–22.1 (encompassing *CARD11*, *MACC1*, *RAC1* and *UNCX*; GSD = 0.8150 compared with non-metastasizing primaries, *q* = 2.87 × 10^−4^) and 8q22.1–8q24.1 (encompassing *UBR5*, *CDH17* and *MYC*; GSD = 0.5232, *q* = 1.85 × 10^−2^; Extended Data Fig. 7a,b).

Taken together, these data suggest that metastasizing clones are larger than non-metastasizing clones in the primary tumour, probably reflecting a fitness advantage over their non-metastasizing counterparts.

## Discussion

We present the results of TRACERx, a longitudinal study tracking the evolution of early-stage NSCLC through space and time, representative of real-world experience within a universal healthcare system. The study design highlighted the importance of both primary and metastatic tissue sampling when interpreting the timing and mode of metastatic divergence. We find that approximately 75% of metastases diverge late, after the last clonal sweep in the primary tumour and that the majority of primary clonal mutations, and indeed driver mutations, persist in the metastases, consistent with previous results^23^. By contrast, other studies, including in breast and colorectal cancer, have found predominantly early divergence^24–26^. This could be confounded by undersampling of the primary tumour or by using region/sample-based rather than clone-based phylogenetic reconstructions^24–26^. Indeed, it is clear that there are no standardized methods or definitions for the assessment of timing of divergence or modes of dissemination, meaning that we need to interpret comparisons across studies with caution^24,27–33^.

Our simulations suggest that, for early divergence cases (32 out of 126 sequenced TRACERx metastatic cases), the metastatic clone would have likely arisen when the primary tumour diameter was less than the typical size threshold (at least 8 mm) used to guide further investigations in modern solid nodule management protocols^10–16^, potentially limiting the use of computed tomography screening in these tumours. Similar findings have been described in colorectal cancer^26^ and other cancer types^29,34^. Notably, we find that early divergence was significantly associated with smoking status at the time of primary tumour surgical resection, suggesting that smoking may provide the fuel for ongoing clonal sweeps after metastatic divergence, enabling cancer cells to continually adapt to their environment. Consistent with previous findings, we also observed that platinum chemotherapy acts as a potent mutagen and contributes to tumour heterogeneity and evolution^6–8^.

Consistent with previous work^23,35^, we observed predominantly monoclonal dissemination of metastases (68% of cases), with the remainder exhibiting polyclonal dissemination. The number of monoclonal dissemination cases is highly likely to be an overestimate owing to sampling of a limited number of metastases. Monoclonal dissemination suggests that metastatic potential was probably acquired once; alternatively, it may reflect ongoing selection or genetic drift within the metastasis, whereby a single clone expands in an originally polyclonal metastasis. Conversely, polyclonal polyphyletic dissemination indicates acquisition of metastatic potential early in tumour evolution or separate clones individually acquiring metastatic potential, or a role for clone–clone cooperation in the metastatic cascade. We also found that polyclonal dissemination at the case level was associated with extrathoracic disease recurrence. In the accompanying Article, we noted that polyclonal dissemination as identified by analysis of circulating tumour DNA, was associated with poor overall survival outcomes^19^. The increased diversity associated with polyclonal dissemination may enable more rapid adaptation to extrathoracic environmental niches and subsequent heterogeneous treatment responses between metastases, providing a possible mechanism accounting for this survival difference. We find that less than 20% of primary LN metastases seed recurrent/progressive disease, suggesting that primary LN metastases are usually a hallmark of metastatic potential rather than a gateway to metastases. Similar findings have been noted in breast, oesophageal, prostate, colorectal and lung cancer^27,33,36–38^. We also find evidence for recurrence/progression samples seeding other recurrence/progression samples, a phenomenon that has been demonstrated in other tumour types^18,24,39,40^.

Paired analysis of multiregion primary tumours and metastases revealed that the metastatic seeding clones appeared fitter than their non-seeding counterparts: they occupied larger areas within the tumour with evidence of selection of driver alterations in lung cancer genes. This was particularly marked in LUSC, where positive selection was observed only in seeding clones. These results may provide the biological mechanism underpinning the findings in the accompanying Article, that tumours with a large recent subclonal expansion in at least one region were associated with poor disease-free survival^17^. Overall, we identify two categories of somatic alterations involved in the metastatic transition. Certain somatic alterations, including *MDM2* amplification in LUAD and *TP53* mutations in LUAD and LUSC, were almost always truncal and maintained, occurring before metastatic divergence, and associated with an increased propensity for metastasis. By contrast, amplification of *HIST1H3B* in LUAD was frequently absent/subclonal within the primary tumour, and may therefore confer increased metastatic potential to a minority of cells or selective advantage in their new metastatic niche.

These data raise the potential for evolutionary measures of tumour biology to forecast metastatic outcome and drive precision treatments specific to emergent metastasizing clones in the adjuvant setting. They highlight the need for research autopsy programs, such as PEACE (Posthumous Evaluation of Advanced Cancer Environment; ClinicalTrials.gov: NCT03004755), which enable extensive sampling of metastases to infer clonal relationships, dissemination patterns, and inter- and intrametastatic heterogeneity with greater accuracy, as well as the need for dynamic and continuous temporal assessments of disease evolution. Indeed, it is not usually possible to acquire multiple biopsies throughout a patient’s treatment journey, and non-invasive methods, such as circulating tumour DNA analyses to track the emergence of seeding clones will be vital to help us better understand the biology of disease progression^41–43^.

## Methods

### The TRACERx 421 cohort

The TRACERx study (https://clinicaltrials.gov/ct2/show/NCT01888601) is a prospective observational cohort study that aims to transform our understanding of NSCLC, the design of which has been approved by an independent research ethics committee (13/LO/1546). Informed consent for entry into the TRACERx study was mandatory and obtained from every patient. All patients were assigned a study identity number that was known to the patient. These were subsequently converted to linked study identities such that the patients could not identify themselves in study publications. All human samples (tissue and blood) were linked to the study identity number and barcoded such that they were anonymized and tracked on a centralized database, which was overseen by the study sponsor only.

The cohort represents the first 421 patients whose primary tumour and metastatic samples were received for processing, who met the eligibility criteria as outlined in ref. ^17^ and from whom collected tumour samples could be sequenced prospectively according to the filtering steps outlined in the CONSORT diagram (CONSORT flow chart; Extended Data Fig. 1).

### Sample processing

#### Sample extraction and sequencing

##### Fresh frozen

Sample extraction and sequencing for fresh frozen samples is summarized in the accompanying Article^17^. Where smaller samples were acquired (for example, core or endobronchial ultrasound guided biopsies), multiregion sequencing was not performed. For sequencing of fresh frozen recurrence/progression samples, paired germline DNA was resequenced in the same run, using germline DNA from aliquots extracted at recruitment.

##### FFPE

For every formalin-fixed, paraffin-embedded (FFPE) tissue block, 2 × 20 μm sections of Cresyl-Violet stained slides were acquired and mounted onto Leica glass slides with a polyethylene naphthalate membrane (4 μm, 27 × 76 mm), sandwiching a 5 μm haematoxylin and eosin slide, which was used to guide dissection. The area was marked by a histopathologist, and any lesions of less than 3 mm in diameter underwent laser-capture microdissection, with larger lesions undergoing macrodissection with a sterile scalpel.

DNA was extracted within 48 h of micro/macrodissection using the Qiagen GeneRead FFPE DNA kit according to the manufacturer’s protocol. This kit contains the UNG (uracil-*N* glycosilase) to minimize FFPE-associated C > T artefacts. The DNA was quantified (Qubit; Invitrogen) and quality-assessed (TapeStation; Agilent technologies) and only samples with a DNA integrity number of greater than 2 were used for downstream processing. The samples were mechanically sheared using the Covaris instrument in a 0.1 mM EDTA buffer solution. Libraries were prepared using 50–200 ng of sheared DNA as input for a modified version of the KAPA HyperPrep library preparation kit (Roche). Modifications included the incorporation of the Agilent SureSelect XT oligo adapters and primers. The remainder of the protocol was performed according to the fresh frozen TRACERx WES sequencing pipeline, with 7–9 PCR cycles used to amplify the DNA to the required 750 ng for hybridization. Sequencing was performed as for the fresh frozen samples, although no additional germline sequencing was performed.

### Bioinformatics pipeline

The bioinformatics pipeline, including quality-control checks, filtering of low confidence variants and phylogenetic reconstruction, used for data analysis is summarized in the accompanying Article^17^. When combining the primary tumour and metastasis regions, the resulting mutation calls and somatic copy-number segmentation may differ from the output of analysing the primary tumour regions alone. These changes could affect downstream analyses, including WGD calls, mutation clustering and phylogenetic tree reconstruction. Similar to the accompanying Article, unless otherwise specified, we limit our phylogenetic-based analyses to the default tree topology, even if multiple tree solutions were reconstructed.

For FFPE samples, modifications to the somatic copy-number aberration detection pipeline were incorporated to address the increase in the fluctuations seen in FFPE-sample logR segmentation. The mean logR value for all SNPs within a BAF segment was assigned as the segmented logR value for that BAF segment. Many small segments remained after this adjustment. These small segments corresponded to logR segments that do not have heterozygous SNPs within them and, therefore, no corresponding BAF segments. Each of these non-BAF segments was subsequently compared to its preceding or following segment within the same chromosome, and joined to the segment with the closest mean logR value until there were no logR-only segments present. The overall mean logR in the newly joined segments was recalculated and used for downstream analyses. Finally, segments corresponding to the lowest logR values (<5% of the sample) were removed.

### Analysis

#### Timing divergence

##### Phylogenetic-based definitions

Timing of divergence was performed relative to the last clonal sweep in the primary tumour. A summary of how individual mutation clusters were defined as clonal, subclonal and absent in individual tumour regions can be found in our accompanying Article^17^. Briefly, clusters that were clonal in all regions of interest (i.e. all primary regions, or all metastatic samples) were defined as clonal within the primary or metastases, respectively. Clusters that were subclonal or absent from at least one region of interest were defined as subclonal, while clusters that were absent from all regions of interest were defined as absent at the tumour level. The total number of mutations associated only to clusters defined as clonal across all primary tumour regions was calculated. For each metastatic sample, the total number and proportion of primary-clonal mutations that were also clonal in the metastasis was computed. If this proportion was less than one, meaning that not all primary-clonal mutations were defined as clonal in the metastatic sample, the metastasis was classified as early diverging. By contrast, if all primary-clonal mutations were clonal within the metastasis, the metastasis was defined as late diverging.

If multiple metastatic sites were sampled for a patient, the case-level classification of the timing of divergence was performed analogously by estimating the metastasis-level clonality. Thus, if all metastatic samples were defined as late diverging, the overall classification would also be late divergence, whereas, if at least one metastatic sample was defined as early diverging, the overall timing would also be early.

##### Region-based presence/absence of mutations

An orthogonal region-based approach was used to define the mutations present in all primary tumour regions (primary-ubiquitous). All mutation loci overlapping genomic segments of LOH in any region were filtered out.

Similar to the phylogeny-defined method, the proportion of primary-ubiquitous mutations shared with the metastatic samples was calculated. This proportion was compared in the phylogeny-defined early- and late divergence cases.

##### LOH-based definitions

The timing of divergence of metastases was also examined using LOH. If a primary tumour clonal LOH event occurred (that is, lost in all cells in the primary tumour or is ubiquitously lost in the primary tumour), a metastasis that does not demonstrate the same LOH event must have diverged earlier as such events cannot be regained later in tumour evolution.

Allele-specific arm-level LOH events were defined as primary-ubiquitous if the same allele was lost in all primary tumour regions. Arm-level loss was defined as ≥75% of the chromosome arm being lost. The proportion of primary-ubiquitous LOH events shared in the metastases was compared in the phylogeny-defined early and late divergence cases.

##### WGD-based definitions

Primary tumours with a clonal WGD (that is, the same WGD event in all primary regions^17^) were identified and the WGD status of the paired metastases was explored. A metastasis was defined as diverging early if no WGD was seen in the metastasis, or a separate WGD event was identified. Metastases were defined as having diverged late if the same WGD event detected in the primary tumour regions was identified in the metastases.

#### Sampling bias

To determine the effect of primary-tumour sampling bias on the timing of metastatic divergence, all cases defined as late divergence were considered. For each such case, given *n* primary regions, all possible combinations of primary tumour region downsampling were considered between 1 and *n-1* regions.

For each single region, the clonal clusters defined in the single region were considered and the proportion of shared clonal mutations between the single region and the metastases was calculated, as described above.

Similarly, when downsampling to two regions, all possible combinations of two out of *n* regions were considered and the percentage of clonal mutations, as defined across the two regions, shared with the metastases was calculated. Finally, the average percentage of shared clonal mutations was computed across all possible combinations to determine the timing of divergence.

This approach was repeated until *n* −1 regions were considered, and the average proportion of shared clonal mutations as well as the classification of the timing of divergence were highlighted.

#### Signature detection

Mutations private to the recurrences or progression samples were fit to deconstructSigs (v.1.9.0)^44^. Mutation counts were normalized using the ‘exome2genome’ parameter within the package. COSMIC Mutational Signatures v.3.2—in particular, SBS1, SBS2, SBS4, SBS5, SBS13, SBS17b, SBS18 and SBS92, which are signatures found to be active in lung cancer genomes^45^, and SBS31 and SBS35, related to cisplatin exposure^6,46^—were used to reconstruct the mutational profiles. Only samples with more than 50 mutations were included. Thus, of the 67 recurrence/progression samples from 48 patients, only 20 samples from 19 patients were included.

#### Modelling

A previously existing agent-based model of tumour growth and evolution^9,47^ was adapted to simulate the timing and mode of metastasis divergence. In brief, the original model simulates the growth of a tumour through the division of individual cells which accumulate mutations at a set mutation rate. The tumour grows in populations or ‘demes’ of 5,000 cells until it reaches a size of 10^9^ cells, when the simulation stops. The simulated tumour is then ‘sampled’ in 8 regions of around 50,000 cells. For each region, exome sequencing is simulated taking into account sequencing error rates for standard Illumina short read sequencing and a mean depth of coverage of 400×, similar to that used in the sequencing of the TRACERx cohort. The simulation produces a file with the minor allele frequency of the detected mutations in each sample.

The model used here was modified from the original to include a dynamic selection landscape. Each individual cell has a fitness value associated with it, which controls its probability of dividing. A cell will divide if its fitness divided by the maximum fitness in the deme is larger than a random number between 0 and 1 drawn from a uniform distribution. Cells with large fitness values will therefore be more likely to divide than those with lower values. Moreover, division will be more likely in demes with low populations and will become increasingly unlikely as the deme approaches its population limit of 5,000 cells. Given that the growth rate is a combination of the division and death rates, the death rate was fixed to avoid further increasing the stochasticity of the model. The death rate of 0.2 was chosen so that the modeled mutation burden was comparable to the mutation burden observed in the TRACERx cohort.

The fitness effect of each mutation is drawn from a distribution of fitness effects (DFE) defined by an asymmetric Laplace distribution centred around 0, and skewed towards negative values, based on the DFE measured in different somatic evolution systems^48–50^. The global selection coefficient defined the mean of the exponential distribution of negative fitness effects, whereas the mean of the exponential distribution of positive fitness effects was half this value. The global selection coefficient therefore controls the spread of the DFE. Furthermore, the possibility of driver mutations was added where a mutation could have a positive fitness effect 10 times larger than the global selection coefficient with a probability of 10^−5^, the mutation rate of driver mutations for somatic evolution in cancer^51^. A global selection coefficient set to 0.01, the maximum selection coefficient used in all simulations, would result in a DFE for normal mutations ranging from −0.07 to 0.02, with low probability driver mutations with a fitness effect of around 0.1. These values are similar to those observed in somatic evolution when selection is measured as the relative increase in growth rate^49,52^. A high global selection coefficient would result in broader DFE distributions and, therefore, more intense selection, whereas a global selection coefficient of 0 would result in neutral evolution, in which none of the mutations have a fitness effect. We also accounted for the fitness effect of large genomic events. The DFE for such events is less well defined but their fitness effects are likely to be vast, given that such events can affect multiple genes at once^53^. To account for these events, a DFE broader than that used for mutations was defined, whereby the mean of positive fitness effects was twice that of mutations, and three times larger for negative effects. These events therefore had the potential to result in highly positive or negative fitness effects. The probability of such events taking place was set at 0.3 per cell division based on observed rates of genome mis-segregation during cell division^54,55^. Only cells that had acquired a specific mutation enabling structural rearrangements were affected.

To simulate metastases, cells were randomly taken from the cell surface to seed a new tumour^26^. The cells were sampled at different primary tumour sizes, and from one or three regions of the primary tumour. Moreover, one or multiple seeding cells were taken from each primary region.

To obtain measures of timing of divergence, mutations that had a variant allele frequency of above 0.3 in 90% of all regions sampled from the primary tumour were considered to be clonal in the primary tumour. Primary–metastatic pairs were considered to be late if all primary clonal mutations were present in the metastatic tumour, and early otherwise, similar to the approach used in the sequencing data. All simulations were run with a selection coefficient of 0.01 both in the primary and metastatic tumours. To examine the mode of dissemination from different seeding patterns, the metastases were seeded from either 1, 10, 30 or 100 cells from either one or three regions of the primary tumour. The primary tumour was always run under a selection coefficient of 0.01, whereas metastatic tumours were run under selection coefficients of either 0, 0.001, 0.005 or 0.01. The resulting variant allele frequency files from the simulations were then formatted to be run through the same PyClone pipeline used to infer dissemination modes from the sequencing data.

All simulations were run for mutation rates of either 0.4 or 0.6 mutations per division per base pair (bp) in the exome (6.6 × 10^−9^ and 10 × 10^−9^ bp per division, respectively) on the basis of observed mutation rates in lung cancer^56^. Twenty replicates of simulated primary–metastatic pairs were run for each combination of parameters.

Cell volume was calculated assuming a cubic cell with a side of 15 μm, the typical diameter for a parenchymal cell^57^. Total tumour volume was calculated as the individual cell size multiplied by the number of cells in the tumour. A percentage of the total tumour cells in the tumour were added to account for purity.

#### Classifying dissemination patterns

Within each primary tumour, we identified which cancer clone(s) were involved in metastatic dissemination and classified the dissemination pattern as monoclonal, if only a single clone of the primary tumour seeded metastatic tumours, or polyclonal, if multiple cancer clones were involved in seeding. Specifically, for each individual metastatic sample, if all mutation clusters shared between the primary tumour and metastasis were found to be clonal within the metastasis, the dissemination pattern was defined as monoclonal. Conversely, if any cluster defined as subclonal within the metastatic sample was also present in the primary tumour, the divergence was classified as polyclonal.

If only a single metastatic sample was considered for a case, the case-level dissemination pattern matched the metastasis level dissemination pattern. If multiple metastases were sampled and the dissemination pattern of any individual metastatic sample was defined as polyclonal, the case-level dissemination pattern was also defined as polyclonal. Conversely, if all metastatic samples followed a monoclonal dissemination pattern, all shared clusters between the primary tumour and each metastasis were extracted. If all shared clusters overlapped across all metastatic samples, the case-level dissemination pattern was classified as monoclonal, whereas, if any metastatic sample shared additional clusters with the primary tumour, the overall dissemination pattern was defined as polyclonal.

Furthermore, the origin of the seeding clusters was determined as monophyletic if all clusters appear along a single branch, and polyphyletic if clusters were spread across multiple branches of the phylogenetic tree. Thus, if a metastasis was defined as monoclonal, the origin was necessarily monophyletic. For polyclonal metastases, the clusters were mapped to branches of the evolutionary tree. If multiple branches were found, the origin was determined to be polyphyletic, whereas, if only a single branch gave rise to all shared clusters, the origin was defined as monophyletic.

For case-level definitions, a similar approach was used. If any metastasis was defined as polyphyletic, the overall origin was also defined as polyphyletic. Conversely, if all metastases were monophyletic in origin, all branches containing shared clusters were counted. If only a single such branch existed, the case-level origin was classified as monophyletic.

To account for variation in the topologies of the phylogenetic tree, the classification of origin was performed on every possible tree topology for a given case. If all classifications overlapped, the multitree adjusted origin was defined as the consensus, while cases with differing origins based on the topology were highlighted as uncertain.

#### Defining the seeding clones

The seeding clone is defined as the most recent shared clone between the primary tumour and metastases. Any cluster present in the primary tumour (defined as clonal or subclonal) and absent from the metastases was defined as primary-unique, any cluster present solely in the metastases and absent from the primary tumour was defined as metastasis-unique, while all clusters present in both the primary tumour and metastases were defined as shared.

The shared clusters were mapped to the phylogenetic tree to determine the most recent shared cluster using a leaf-up approach. If the shared clusters could be mapped to a single branch of the phylogenetic tree, the clonality of the most recent shared cluster was determined in the metastasis. If the most recent shared cluster was clonal in the metastasis, this cluster was defined as the only seeding cluster for the metastatic sample. By contrast, if the most recent shared cluster was subclonal within the metastasis, the parent cluster was also considered. This was done iteratively until the first shared cluster that was clonal in the metastasis was found. Clusters along this path were defined as seeding if their phylogenetic CCF^17,58^ (phyloCCF) value was greater than the phyloCCF of the child cluster.

If the shared clusters mapped to multiple branches of the phylogenetic tree, each branch was considered separately in the manner described above. If a parent cluster was shared between multiple branches, CCF values of both branches were added together, and the iterative approach continued until the first cluster was found to be clonal in the metastasis.

#### Inferring metastatic migration patterns

The MACHINA algorithm^18^ (v.1.2) was applied to infer the metastatic migration patterns of distinct tumour clones across the cohort. As MACHINA requires a tumour phylogenetic tree for each patient as input, we provided MACHINA with the default phylogenetic trees reconstructed in this study, and applied MACHINAs pmh_tr function, which infers the most parsimonious migration histories with tree polytomy resolution^18^. Furthermore, MACHINA requires as input clone proportions, that is, the proportions of cancer cells belonging to each tumour clone present at the time of sampling in each tumour region. As such, we estimated clone proportions in each region by using the estimated mean phyloCCF value across the related mutation clusters. To do this, we developed a bottom-up iterative algorithm that estimates clone proportions starting from the leaves of the tumour phylogenetic tree. Specifically, the clone proportion of each mutation cluster corresponding to a leaf of the phylogenetic tree was estimated to be equal to its phyloCCF, as the corresponding mutations were inferred to be present only in the cells belonging to its related clone. For every ancestral mutation cluster, the clone proportion of the corresponding clone was inferred by calculating the difference between the phyloCCF of the mutation cluster and the sum of the phyloCCFs of all its descendants. For example, if the leaf cluster had a phyloCCF of 1 in a region, no other clusters in the phylogenetic tree were present as clones. However, if a leaf cluster had a phyloCCF of 0.75, some parental clusters along the tree were inferred to have a clone proportion summing to 0.25. As phyloCCF is a point estimate of the corresponding underlying parameter, the phyloCCF of mutations that were inferred to be clonal in a tumour region might be generally different to 1. Since these deviations might affect the estimation of clone proportions, we corrected the mean phyloCCF of every clonal cluster to be exactly equal to 1.

The estimated clone proportions were used to create a clone tree, which was used as an input to MACHINA to infer metastatic migration patterns. Specifically, MACHINA was run by specifying the primary lung tumour and implementing each metastatic tumour as a separate site. Moreover, MACHINA was run considering all of the possible assumptions about the possible migration patterns that can be evaluated (parallel single source seeding, single source seeding, multi-source seeding, reseeding). To explore seeding of one metastasis by another site, the results from the single-source seeding output from MACHINA were used, as these provide the most conservative results of MACHINA.

In addition to exploring the different routes of metastatic dissemination, the results of MACHINA can be used to identify metastatic seeding clones. Thus, to provide further evidence to the identified seeding clones, we compared the results of MACHINA with those inferred by the new method in this study. Under the parallel single-source seeding assumption adopted in this analysis, we considered only the results of MACHINA using the same dissemination model. Moreover, the definition of monoclonal and polyclonal seeding from MACHINA does not take into account the tree, as done in this study. Thus, whereas MACHINA defines cases as polyclonal only if at least one metastasis sample is polyclonal, cases with a single monoclonal or multiple monoclonal metastases are both defined as monoclonal. To reconcile these differences, we adapted a similar definition: all cases that we define as polyclonal but that have multiple monoclonal metastases were redefined as monoclonal for this comparison.

#### Calculating the clonal dispersion index

The clonal dispersion index was calculated as follows. For a tumour with *n* regions, subclonal cluster dispersion of each cluster *i*, with CCF *x*_*i*_, was calculated as:

$$D=1-\frac{\max \left({p}_{i}\right)-\frac{1}{n}}{1-\frac{1}{n}},$$

Where ${p}_{i}=\,\frac{{x}_{i}}{{\sum }_{i=1}^{n}{x}_{i}}$ is the vector of CCF proportions. Each subclone was therefore given a score from 1, indicating the clone was evenly spread across all regions, to 0, where the clone was entirely unique to a single region. We compared the maximum CCF and subclonal dispersion to investigate both how dominant in any region and spread out across the regions the clusters were to quantify subclonal expansion.

#### d*N*/d*S* analysis

##### Cohort level

An adapted version of the dNdScv method (v.0.0.1.0)^20^ was used to estimate global d*N*/d*S* values. In this adapted version, the global rates were estimated using all mutations (similar to running the original dNdScv function without specifying a gene list). Subsequently, the inferred global rates were used to estimate the global d*N*/d*S* estimates for a curated set of lung cancer genes. This list was formed of lung cancer genes as described in refs. ^3,20,21,59^, which were subsequently filtered based on expression in the TRACERx 421 cohort (median transcripts per million (TPM) > 0.2). This approach was run separately on mutations found in the seeding cluster and primary-unique mutations, as well as on subclonal mutations of non-metastatic primary tumours, as well as for LUAD and LUSC.

##### Gene level

The dNdScv function was run on mutations associated with the seeding clusters, as well as on the combination of mutations classified as primary-unique and subclonal mutations of non-metastatic tumours, for a curated set of lung cancer specific genes. This list was formed of lung cancer genes as described in refs. ^3,20,21,59^, which were subsequently filtered based on expression in the TRACERx 421 cohort (median TPM > 0.2).

The d*N*/d*S* point mutation estimate was calculated by combining the d*N*/d*S* estimates of missense and truncal mutations. The odds ratio of each gene was computed as the d*N*/d*S* estimate within the seeding mutations divided by the d*N*/d*S* estimate within the combined primary-unique and non-metastatic mutations. If the odds ratio was >2, the gene was classified as seeding favoured; if the odds ratio was <0.5, the gene was classified as primary favoured; and, otherwise, the gene was classified as primary and seeding favoured. The results were plotted for all genes with global *q* < 0.1 as calculated by dNdScv.

This analysis was performed separately for LUAD and LUSC tumours, as well as by combining both histological subtypes.

To statistically compare d*N*/d*S* values across the two groups (seeding mutations versus combined primary-unique and non-metastatic mutations), a published approach outlined in ref. ^60^ (https://zenodo.org/record/3966023#.YanjS_HP2cZ) was used (variable_dNdS_twodatasets). This approach compares d*N*/d*S* ratios of two datasets using a likelihood-ratio test. For a given gene *g*, the one-sided test uses the following null and alternative hypotheses^60^:

H_0_: *ω*_*g*,1_ ≤ *ω*_*g*,2_

H_1_: unconstrained *ω*_*g*,1_ and *ω*_*g*,2_

Where *ω*_g,i_ is the dN/dS estimate for gene *g* in dataset *i*. This approach corrects for differences in mutation density due to coverage or mutational signatures, as well as removes the effect of global differences in d*N*/d*S* ratios across the genes.

Therefore, dNdScv was run on the two datasets (seeding mutations := mutations from seeding clusters; non-seeding mutations := mutations from primary-unique clusters and mutations from non-metastatic tumours) independently. All genes with *q* < 0.1 as calculated by dNdScv were selected from both datasets and used for subsequent comparison. To calculate which genes were significantly enriched in seeding mutations, the function variable_dNdS_twodatasets was applied to seeding mutations as dataset 1 and non-seeding mutations as dataset 2 using the genes that were significant (*q* < 0.1) in the seeding mutations. Conversely, to calculate which genes were significantly enriched in non-seeding mutations, the function variable_dNdS_twodatasets was applied to non-seeding mutations as dataset 1 and seeding mutations as dataset 2 using the genes that were significant (*q* < 0.1) in the non-seeding mutations. For both analyses, multiple-testing correction (BH) was performed for the final list of significantly enriched genes.

#### Paired mutation analysis

Each mutation cluster was classified as metastasis favoured if it was absent in the primary and subclonal or clonal in the metastasis, or subclonal in the primary and clonal in the metastasis; primary favoured if it was clonal in the primary and subclonal or absent in the metastasis, or subclonal in the primary and absent in the metastasis; and maintained otherwise. The mutation cluster definition was then applied to each mutation within that cluster. The cohort was separated into LUAD and LUSC.

First, non-driver mutations were used to calculate the ‘background’ rate of metastasis favoured, primary favoured and maintained mutations. Subsequently, the number of metastasis favoured, primary favoured and maintained driver mutations was calculated for each gene containing at least 5 driver mutations and compared to the background proportion of non-driver mutations.

This was used to estimate the proportions of metastasis favoured, primary favoured and maintained mutations using a multinomial test; *P* value correction using the Benjamini–Hochberg^61^ method was subsequently performed.

#### Unpaired SCNA analysis

To identify genomic regions that demonstrated a significant SCNA positive-selection score at each genomic location, GISTIC2.0 (v.2.0.23)^22^ was run on the following two cohorts independently to produce SCNA positive-selection scores (G-score values), treating LUAD and LUSC separately: primary tumour samples from non-metastatic patients, excluding patients that presented with LN metastases at surgery; and metastasis samples from recurrent patients, including primary LN metastases.

GISTIC2.0 takes as input a copy-number profile across the genome from one sample per patient. To investigate genomic regions of recurrent amplifications (/losses and deletions, respectively), we constructed the single-sample copy number profile for each tumour by selecting the maximum (/minimum, respectively) ploidy-corrected total copy number per segment across the genome.

To compare the GISTIC2.0 output between the metastasis and non-recurrent primary cohorts, we compared the *G*-score of all genes between the two cohorts. To measure the *G*-score per gene, we matched overlapping GISTIC2.0 segments with gene genomic positions. For genes that did not overlap any GISTIC2.0 output segments, we used the mean *G*-score of the two neighbouring segments. We then investigated oncogenes and tumour suppressor genes from our curated driver gene list in amplifications and losses, respectively, taking forward those that were found to have significant *G*-scores in our metastasis cohort for further analyses. For these genes, we calculated the difference in G-score values (G-score difference, GSD) between the metastasis and non-recurrent primary cohorts, to measure the difference in positive selection at these loci for the two cohorts.

When performing the unpaired SCNA analyses separately for primary LN/satellite lesions and recurrence/progression samples, we constructed a single copy number profile for each sample type (that is, primary tumour, primary LN/satellite lesions and recurrence/progression samples), and performed comparison analyses as described above.

#### Paired SCNA analysis

Using the driver genes found to be significantly recurrent in the unpaired analyses, we performed paired analyses of metastasizing primary tumour regions and their matched metastases to determine where in the metastatic transition these events had occurred. We first classified the copy number status of all segments overlapping these genes in the matched primary–metastasis cohort as lost or amplified relative to the sample ploidy^62^. Next, for tumours that had an event in a gene in at least one sample, we classified the event as primary favoured, metastasis favoured or maintained: if the event was present in both metastasizing primary regions and matched metastases, it was classified as maintained; if the event was present in metastasizing primary regions but absent from matched metastases, it was classified as primary favoured; and finally, if the event was absent from the metastasizing primary regions but present in the matched metastases, it was classified metastasis favoured. For each driver gene with an event present in at least five tumours, we then performed a multinomial test to determine whether the number of event classifications in this gene was significantly different compared to the background proportion of maintained, metastasis favoured and primary favoured classifications in all non-driver genes.

When performing the above paired SCNA analysis separately for primary LN/satellite lesions and recurrence/progression samples, we considered only patients whose set of metastatic samples were either all primary LN/satellite lesions or all recurrence/progression samples.

#### Depiction of clonal structure in tumour samples using clone maps

In Figs. 3 and 4, we depict the CCFs of subclones estimated using our WES pipeline accounting for the nesting structure determined by phylogenetic tree building. These depictions were generated using the cloneMap R package^63^ (v.1.0.0), which is available at GitHub (https://github.com/amf71/cloneMap).

### Statistical information

All statistical tests were performed in R (v.3.6.3 and 4.1.1). No statistical methods were used to predetermine sample size. Tests involving comparisons of distributions were performed using two-sided Wilcoxon tests (‘wilcox.test’) using paired or unpaired options where appropriate. Tests involving comparison of groups were performed using two-sided Fisher’s exact tests (‘fisher.test’). Hazard ratios and *P* values were calculated using the survival package (v.3.2.13). For all statistical tests, the number of data points included is plotted or annotated in the corresponding figure; and all statistical tests were two-sided unless otherwise specified.

### Reporting summary

Further information on research design is available in the Nature Portfolio Reporting Summary linked to this article.

## Online content

Any methods, additional references, Nature Portfolio reporting summaries, source data, extended data, supplementary information, acknowledgements, peer review information; details of author contributions and competing interests; and statements of data and code availability are available at 10.1038/s41586-023-05729-x.

## Supplementary information

Supplementary Note Orthogonal implementation for dissemination.

Reporting Summary

## Figures and Tables

**Extended Data Table 1:**
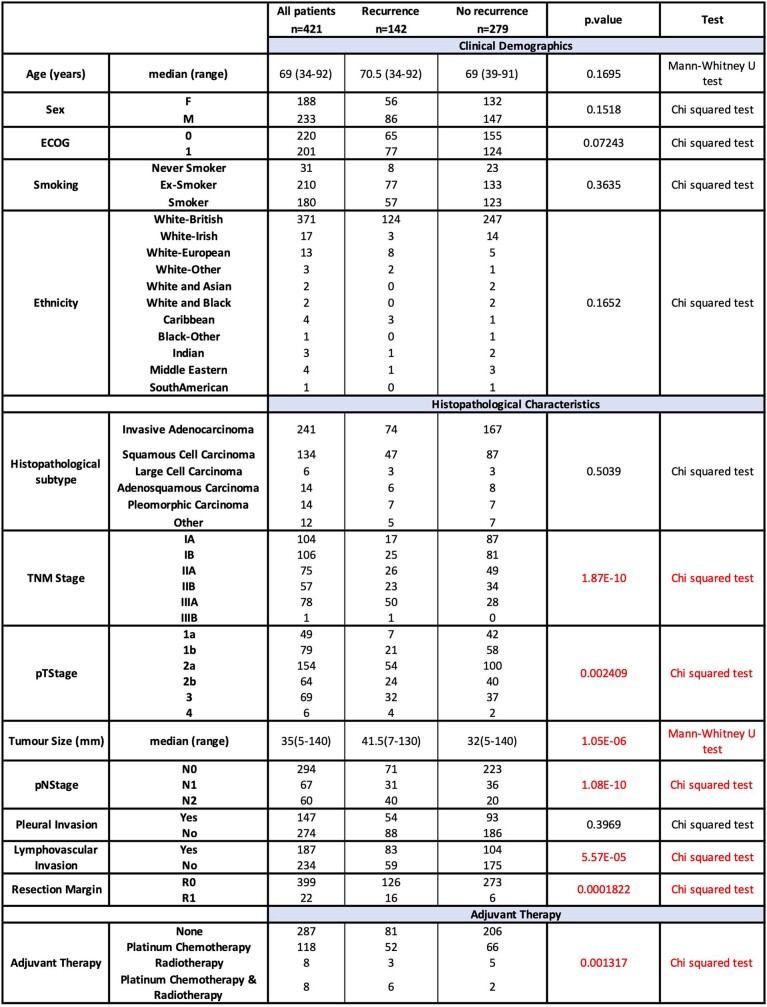
Clinical and histopathological characteristics of the TRACERx 421 cohort

Comparison of baseline clinical and histopathological characteristics of patients who develop recurrent disease versus those who do not. All significant (p < 0.05) results are shown in red with corresponding statistical tests used. All tests performed were two-sided. F: Female, M: Male; ECOG, Eastern Cooperative Oncology Group performance status.

**Extended Data Table 2:**
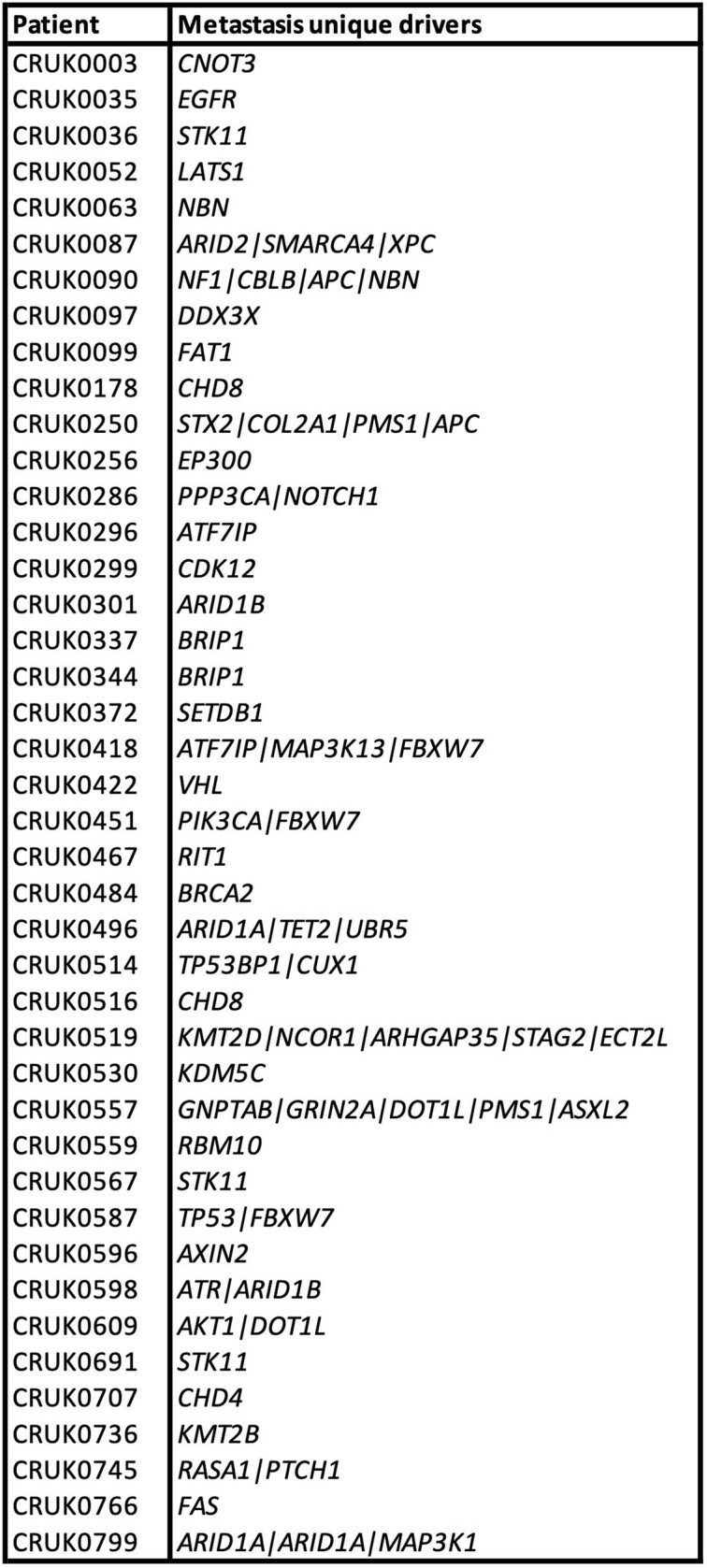
Overview of metastasis-unique drivers

Where a gene is listed twice, multiple predicted driver alterations within the same gene were identified.

**Extended Data Table 3:**
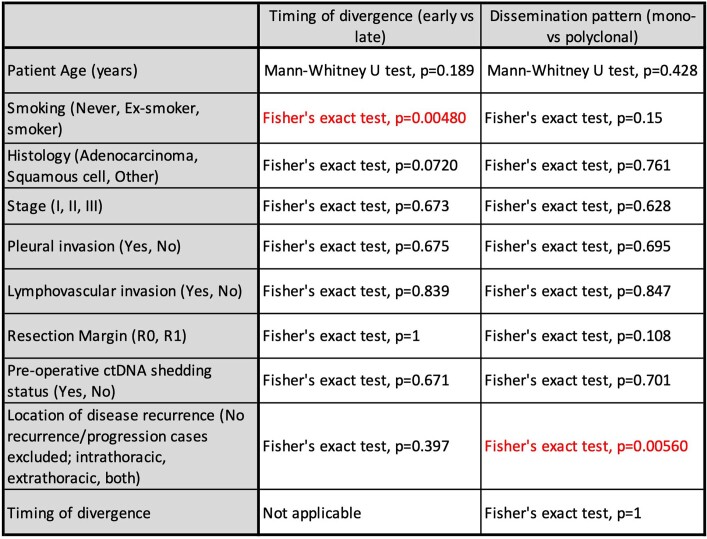
Clinical Associations with timing and modes of metastatic divergence

Age, smoking status, histology, disease stage, pleural and lymphovascular invasion, resection margin, presence of pre-operative circulating tumour DNA (ctDNA) and location of disease recurrence were explored. Early divergence was associated with being a smoker, and polyclonal dissemination was associated with extrathoracic disease recurrence. All significant (p < 0.05) results are shown in red with corresponding statistical tests used. All tests performed were two-sided.

## Data Availability

The WES data (from the TRACERx study) used during this study have been deposited at the European Genome–Phenome Archive (EGA), which is hosted by the European Bioinformatics Institute (EBI) and the Centre for Genomic Regulation (CRG) under accession code EGAS00001006494; access is controlled by the TRACERx data access committee. Details on how to apply for access are available on the linked page.

## Code availability

All code to reproduce figures is available at Zenodo (10.5281/zenodo.7649257) .
