# Supplementary material for: The evolution of non-small cell lung cancer metastases in TRACERx
Source: Nature. 2023 Apr 12;616(7957):534–42. doi: 10.1038/s41586-023-05729-x (PMC10115651; doi:10.1038/s41586-023-05729-x)
Supplement: Supplementary file 1 — Orthogonal implementation for dissemination. [file 41586_2023_5729_MOESM1_ESM.pdf]

nature portfolio

https://doi.org/10.1038/s41586-023-05729-x

Supplementary information

The evolution of non-small cell lung cancer metastases in TRACERx

In the format provided by the authors and unedited

Supplementary Note

Table of Contents

Orthogonal implementation for dissemination **2**

Orthogonal implementation for dissemination

As an orthogonal approach to characterise dissemination patterns, we implemented the published tool MACHINA[1] (Methods: *Inferring metastatic migration patterns*). We observed that for 90% (114/126) of tumours, the dissemination patterns were concordant with our approach (Extended Figure 5h). For the cases with disparate dissemination classifications (12/126), MACHINA defined the dissemination patterns as polyclonal while our approach defined the cases as monoclonal (Extended Figure 5h). This is consistent with our method being conservative and can be traced back to differing assumptions of the two methods; MACHINA assumes no metastasis specific clones can exist, therefore the clone(s) must have been missed within the primary. This can lead to multiple seeding events being inferred. In fact, when relaxing the hypothesis to allow for seeding from sources other than the primary, all 12 cases (100%) where the classifications differed, were predicted with MACHINA to have a metastasis being seeded by another metastatic region compared to only 6 out of the remaining 114 cases (5.3%). Conversely, our method for determining the mode of dissemination (Methods: *Classifying dissemination patterns*) allows for metastasis specific clones and predicts the seeding event to occur prior to this.

References

1. El-Kebir, M., Satas, G. & Raphael, B. J. Inferring parsimonious migration histories for metastatic cancers. *Nat. Genet.* **50**, 718–726 (2018).
